# Scene Perception–Memory Pairing Extends to the Superior Parietal Cortex

**DOI:** 10.1523/ENEURO.0155-26.2026

**Published:** 2026-09-01

**Authors:** Runhan (Nicole) Tang, Dominika Panek, Leila H. Barkoff, Catriona L. Scrivener, Edward H. Silson, Adam Steel

**Affiliations:** ^1^Psychology Department, University of Illinois Urbana-Champaign, Champaign, Illinois 61820; ^2^Department of Psychology, School of Philosophy, Psychology & Language Sciences, University of Edinburgh, Edinburgh EH8 9JZ, United Kingdom; ^3^Beckman Institute for Advanced Science and Technology, University of Illinois Urbana-Champaign, Urbana, Illinois 61801

**Keywords:** episodic memory, imagery, navigation, scene perception, spatial memory

## Abstract

Visual scene analysis relies on a set of scene-selective regions in the posterior cerebral cortex (OPA, PPA, MPA), with a paired memory-responsive counterpart located in the anterior (LPMA, VPMA) or overlapping (MPMA) cortex. The interaction between these pairs of regions is thought to integrate visual input with mnemonic context. Recently, a fourth scene perception area in the superior parietal cortex (the superior place area, SPA) was identified, with a proposed role in visually guided navigation. Whether this region also has an anterior paired memory region is currently unknown. Across two independent fMRI datasets (total *N* = 24, 14 females) using static or dynamic stimuli and distinct memory tasks, we show that recalling visual scenes evokes robust responses in a region immediately anterior and dorsal to SPA (referred to here as the superior place memory area, SPMA). During resting-state fMRI, SPA preferentially coupled with the other scene perception areas, while SPMA preferentially coupled with the other place memory areas. At the whole-brain level, seed-based connectivity revealed that SPA sits at the confluence of four processing streams spanning regions implicated in egocentric scene perception, map-based navigation, perspective taking, and goal-directed movement. These findings extend the perception–memory motif associated with visual scene processing to a fourth cortical surface. The widespread anatomical coupling between scene perception and memory processes reflects the importance of this interaction for flexible, context-grounded navigation.

## Significance Statement

Prior work has identified an anatomical pairing between cortical regions specialized for scene perception and memory on the lateral and ventral surfaces. This architectural motif is thought to support integration of visual input with environmental context. Using two independent fMRI datasets, we show that a recently identified fourth scene perception area in the superior parietal cortex follows the same organizational principle and that it sits at the confluence of cortical streams linking egocentric vision, spatial memory, and goal-directed movement. This positions the superior parietal cortex as a hub for context-grounded navigation.

## Introduction

As we navigate through the world, we rapidly extract information about our visual surroundings to make informed decisions about where we can move and what affordances are available (Bonner and Epstein, 2017; Dwivedi et al., 2020; Bartnik et al., 2025; Mynick et al., 2025; Zamboni et al., 2026). Prior work has linked these abilities to three scene-selective regions on the lateral, ventral, and medial surfaces of the posterior cerebral cortex: the occipital place area (OPA; Hasson et al., 2002; Dilks et al., 2013), the parahippocampal place area (PPA; Epstein and Kanwisher, 1998), and the medial place area (MPA), also referred to as retrosplenial complex (RSC; Bar and Aminoff, 2003; Epstein et al., 2007a; Silson et al., 2016b). Each scene perception area is thought to play a unique role (Epstein and Baker, 2019; Dilks et al., 2022), with OPA supporting identification of paths and boundaries (i.e., navigational affordances; Julian et al., 2016; Kamps et al., 2016; Bonner and Epstein, 2017; Lescroart and Gallant, 2019; Zamboni et al., 2026), PPA supporting scene categorization and landmark recognition (Marchette et al., 2015; Persichetti and Dilks, 2018, 2019; Sun et al., 2021), and MPA supporting map-based navigation (Wolbers and Büchel, 2005; Auger et al., 2012, 2015; Marchette et al., 2014; Persichetti and Dilks, 2019; Steel et al., 2019). Beyond visual analysis, navigation also depends on integrating the current visual input with memory of our surroundings. A key question is how the brain connects scene perception with visuospatial memory.

Remarkably, the brain appears to connect perceptual and mnemonic processes by placing brain areas involved in visual scene analysis and visuospatial memory in close anatomical proximity. Each scene perception area is flanked by an anterior, memory-responsive counterpart: the lateral, ventral, and medial place memory areas (LPMA, VPMA, MPMA; Steel et al., 2021). Using visual imagery to isolate top–down memory processes, recent studies have shown these anterior regions are selectively active during scene recall (Steel et al., 2021, 2023, 2024b, 2025; Srokova et al., 2022), are more functionally connected to the hippocampus than their paired perceptual counterparts, and show unique sensitivity to visuospatial context during both perception and recall (Steel et al., 2021, 2023). The combination of anatomical position and functional connectivity makes the memory-responsive areas ideally positioned to integrate perceptual and mnemonic representations during navigation.

Recently, a fourth scene perception area in the superior parietal cortex, referred to as the posterior intraparietal gyrus scene-selective site (PIGS; Kennedy et al., 2024) or the superior parietal lobule region (SPL; Yoon et al., 2025), has been described. Here we use the term superior place area (SPA). Like OPA, SPA responds preferentially to egocentric motion through scenes (Kennedy et al., 2024; Yoon et al., 2025, 2026) and is more strongly connected to OPA than to PPA or MPA at rest (Yoon et al., 2025). Interestingly, this area shows enhanced connectivity to motor regions involved in leg versus hand movements compared with other parietal areas (Yoon et al., 2026). These studies collectively suggest that SPA plays a specialized role in visually guided navigation.

Does this new scene-selective site in the superior parietal cortex follow the same posterior–anterior perception–memory organizational motif as the lateral and ventral surfaces? The answer is not obvious. On the one hand, because SPA is specialized for online visual guidance of egocentric movement, it may require little access to stored spatial memories. On the other hand, visually guided navigation is not purely reactive; it involves anticipating paths, planning movements, and integrating current views with prior knowledge of an environment. Because these functions require memory of spatial context, SPA may also have a closely affiliated memory area. In prior work with group analyses, we have observed scene memory-related activity in the superior parietal cortex (Steel et al., 2021, 2023), and another recent study that adopted this localization approach also reported memory-evoked activity in the superior parietal cortex (Han and Epstein, 2026). However, these studies did not localize perceptual SPA, and therefore whether the superior parietal cortex has a set of paired perception and memory areas remains unknown. Observing the same perception–memory pairing seen on the lateral and ventral surfaces would suggest that this motif is a general principle of the cortex devoted to visual scene analysis.

Here we asked whether the scene perception and memory activity in the superior parietal cortex follows a similar topographical arrangement to the lateral and ventral surfaces. Across two independent fMRI datasets using static (Experiment 1) or dynamic (Experiment 2) stimuli and distinct memory tasks, we find that scene recall evokes activity in a memory-responsive region (SPMA) immediately anterior and dorsal to SPA. Furthermore, SPA sits at the intersection of four cortical streams spanning regions implicated in egocentric scene perception, map-based navigation, perspective taking, and goal-directed movement.

## Materials and Methods

### Procedure

**Figure 1.**
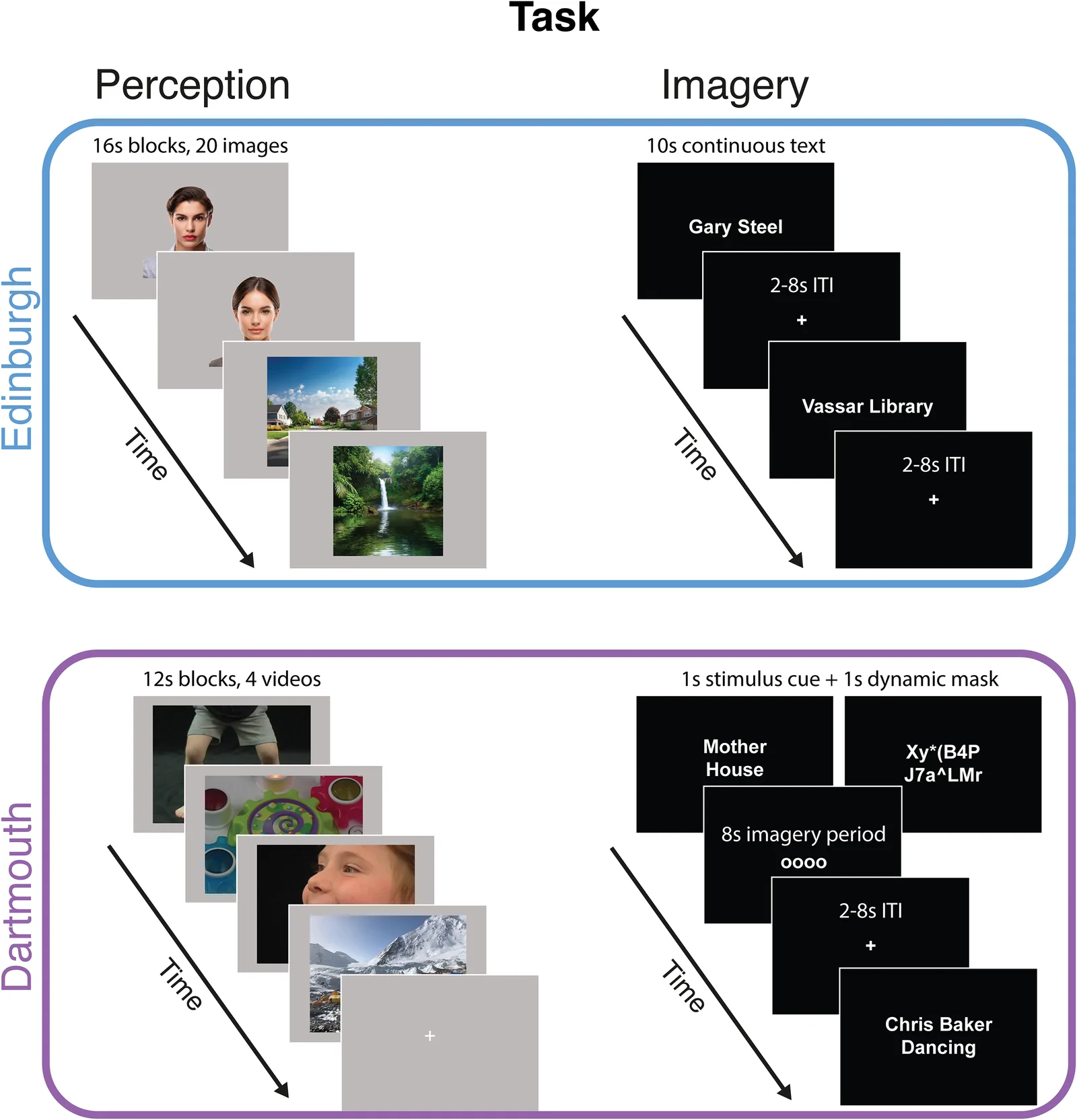
Experimental design. Our study considered two experiments collected at the University of Edinburgh (Experiment 1) and Dartmouth College (Experiment 2). Participants performed perception and memory tasks in both experiments. Top, In Experiment 1, during the perception task, participants saw a series of unfamiliar face and scene images presented in alternating blocks (16 s/block, 20 images; 300 ms on, 500 ms off); during the imagery task, participants imagined photographs of familiar people or place images. In Experiment 2, during the perception task participants saw a series of 3 s videos of unfamiliar bodies, objects, faces, and scenes arranged in blocks (12 s/block) with 12 s fixation cross intermixed; during the imagery task, participants were cued to imagine four different conditions associated with a familiar person: a photograph of the person's face, the person dancing, having a conversation with the person, or looking around a room in the person's house. Participants in the Dartmouth experiment also underwent a 10 min resting-state fMRI scan.

Here, we investigated the topography and connectivity of scene perception and memory processes in the superior parietal cortex in two independent experiments, one collected at the University of Edinburgh and the other at Dartmouth College. We implemented perception and memory tasks in both experiments, which differed in the nature of the stimuli (Fig. 1).

Experiment 1 (Edinburgh) used static images of unfamiliar scenes and faces in the perception task and recall of photographs depicting personally familiar scenes versus people in the memory task. Experiment 2 (Dartmouth) used dynamic scene and face videos in the perception task and recall of familiar scenes versus multiple social stimuli in the memory task. These tasks are described in detail below. To evaluate functional connectivity of the scene perception and memory areas in the superior parietal cortex, participants in the Dartmouth experiment also underwent a resting-state fMRI scan.

### Participants

Data collection was approved by each institution's ethics review boards (protocol numbers: University of Edinburgh, 420–2122/5 and 420–2122/6; Dartmouth College, 31288). All participants gave informed consent to participate. Prior work showed large effect sizes when evaluating the anterior shift of memory and perception on the cortex's lateral, ventral, and medial surfaces (Cohen's *d*s > 1.6; Steel et al., 2021). Based on this effect size (*d* = 1.6; power = 0.8; *α* = 0.05), six participants would be sufficient to detect an effect. Both experiments exceeded this sample size by a factor of two.

#### Experiment 1 (Edinburgh)

This experiment makes use of data reported in Scrivener et al. (2025), which examined group-average activity of category-selective regions during memory tasks. Here, we consider the spatial location of the place memory areas in the posterior cerebral cortex identified in individual participants, including the new area in the superior parietal cortex, which had not been previously analyzed. Twelve participants (mean age, 27; three males) underwent the functional MRI session. All participants were able to perform visual memory based on the Vividness of Visual Imagery Questionnaire (VVIQ, 58.58 mean; range, 38–74). Participants received monetary compensation for their time.

#### Experiment 2 (Dartmouth)

Twelve participants underwent scanning at Dartmouth College (mean age, 30 ± 8.1 years; seven males). All participants reported good imagery ability when asked. Measures of trait-level vividness (i.e., VVIQ) were not acquired. Participants received a gift card as compensation for their time. These data have not been reported previously.

### FMRI tasks

#### Experiment 1 (Edinburgh)

##### Static scene perception localizer

Participants performed two runs of a scene/face localizer task. Participants viewed 20 blocks of 20 unfamiliar scene or face stimuli (images presented 300 ms on, 500 ms off; 16 s per block; 10 × 10° of visual angle) while completing a one-back task. Participants indicated a repeated image by making a right-hand button press when the same image was repeated sequentially, which occurred twice per block. There was no fixation/baseline period between blocks.

##### Scene memory task

Prior to scanning, participants provided photographs of six personally familiar people and six personally familiar places that they could mentally imagine. The names of the individuals/places served as cues for the memory task (visual mental imagery task).

During the imagery task, we instructed participants to create a mental image of the picture linked to the target cue word. This served to standardize their imagery content across stimulus repetitions and encouraged participants to perform imagery rather than recalling general aspects of the people and places. We instructed participants to keep their eyes open and to fixate on the cross during imagery trials.

Participants completed six runs of the mental imagery task, lasting 6 min each. Each imagery cue was presented twice per run (12 total repetitions). One participant only completed four of the six imagery runs.

#### Experiment 2 (Dartmouth)

##### Dynamic scene localizer

Participants performed two runs of a dynamic multicategory visual localizer task. Stimuli were publicly available and used in previous studies (Pitcher et al., 2011) and consisted of 3 s videos of four visual categories: (1) children's faces, (2) body parts (hands, feet, torso), (3) toys, and (4) scenes.

Participants saw blocks of stimuli from each condition, with four movies per block (4 videos * 3 s/video = 12 s/block, 11.4 × 8.5° of visual angle). Participants passively viewed the videos and did not perform any behavioral task. All categories appeared four times per imaging run. We organized runs so that participants saw all categories before any category repeated. Before repeating the category blocks, we included a fixation block (12 s duration) to allow the BOLD signal to return to baseline.

##### Dynamic scene memory localizer

Data from Dartmouth were collected as part of a study investigating spatial and social imagery. Prior to scanning, participants provided the names of four people for whom they could imagine the following four prompts: (1) imagine a room in that person's house, (2) a specific photograph of that person, (3) that person dancing, and (4) having a conversation with that person. These people and prompts served as cues for the spatial versus social imagery task. Here, we only considered the contrast of spatial compared with the mixed social conditions; the results specific to the social imagery conditions will be discussed in a separate report.

In the scanner, we showed participants the names they provided along with one of the following cues: house, photo, dancing, and conversation. This indicated which stimulus they should imagine, and the condition they would perform.

For the imagery conditions, we provided the following instructions to participants:

*House*: Imagine a specific room in that person's house. When you are imagining the room, you should envision that the room has materialized all around you, and you should direct your attention smoothly around the environment as though you were standing in that place.
*Photo*: Imagine a specific photograph of that person. The photograph should only include that person and should focus on their face. Hold that specific photograph in mind as long as the cue appears on screen.
*Dancing*: Imagine that person dancing. You should imagine their body moving dynamically through space in a way that would be natural for them. Focus on their movements and the specific way that they move their body rather than on their face or expression. If it is impossible for you to imagine them dancing, you could imagine them walking, running, or jumping.
*Conversation*: Imagine having a conversation with that person. You should imagine the specific words that they are saying and the sound of their voice. You should not imagine their face or expression, and instead focus on the sound quality, including their tone and unique vocal inflections.

Simulation trials lasted 10 s. Trials began with the name/condition stimulus (1 s), followed by a dynamic mask (1 s, comprising ASCII characters that switched every 200 ms), and the imagery cue (8 s, four circles displayed at the center of the screen). We instructed participants to perform mental imagery for the entire duration that the imagery cue was on the screen. Trials were separated by a jittered ITI (4–8 s), during which time a fixation cross appeared on the screen. During the ITI, we instructed participants to clear their mind and prepare for the next trial.

Participants underwent four runs of fMRI scanning on the social imagery task. Each stimulus × condition pairing was shown once per run (4 stimuli per condition × 4 conditions = 16 trials per run). Each scanning run lasted 280 s (140 TRs).

##### Resting-state fMRI

For participants in the Dartmouth dataset, we collected 10 min of resting-state fMRI data (Biswal et al., 1995). We chose this quantity based on prior work suggesting pairwise regional functional connectivity in cortex stabilizes between 8 and 12 min (Gonzalez-Castillo et al., 2014; Poldrack et al., 2015). During the resting-state scan, we instructed participants to fixate on a white cross shown over a black background and allow their thoughts to wander, keep their eyes open, and avoid falling asleep. After scanning, all participants reported compliance with these instructions.

### MRI acquisition

Data from both sites were acquired on Siemens 3 T Prisma scanners with a 32-channel head coil. Functional scans were acquired using a multiecho multiband echoplanar imaging (EPI) sequence (TR, 2 s; TEs, 14.6, 35.79, 56.98 ms; MB factor, 2; acceleration factor, 2; 48 interleaved slices; phase-encoding anterior to posterior, transversal orientation; slice thickness, 2.7 mm; voxel size, 2.7 × 2.7 mm; distance factor, 0%; flip angle, 70°) identical to prior studies (Steel et al., 2022, 2023, 2024b; Scrivener et al., 2025; Scrivener and Silson, 2025). A high-resolution anatomical image was collected for each participant (Edinburgh, TR, 2.5 s; TE, 4.37 ms; flip angle, 7°; FOV, 256 × 256 × 192 mm; resolution, 1 mm isotropic; acceleration factor, 3; Dartmouth, TR, 2,300 ms; TE, 2.32 ms; inversion time, 933 ms; flip angle, 8°; FOV, 256 × 256 mm; slices, 192; voxel size, 1 × 1 × 1 mm). T1-weighted (T1w) images were segmented, and surfaces were generated using FreeSurfer (version 6.0; Fischl, 2012) and aligned to the fMRI data using align_epi_anat.py and @SUMA_AlignToExperiment (Saad and Reynolds, 2012).

### FMRI preprocessing

#### Experiment 1 (Edinburgh)

MRI scans were processed using AFNI (Cox, 1996), SPM (Friston et al., 1994), FreeSurfer (Fischl, 2012), and SUMA (Saad and Reynolds, 2012). Before preprocessing, the origin of all MRI data was manually reset to the anterior commissure (ac) using SPM display (SPM12). For the structural images, the ac origin was defined in the T1w image and the transformation applied to the T2w image to ensure that they were aligned to each other. For the EPIs in each run, the origin was defined from the first TR of the second echo of the first run and applied to all other echoes and scans in the same session. This procedure was to facilitate coregistration of EPIs and structural scans across the two sessions, given that the origins of the acquired data could be far apart in space.

During preprocessing, five dummy scans were first removed from the start of each run (AFNI 3dTcat). Large deviations in signal were removed (3dDespike; Jo et al., 2013) followed by slice time correction (3dTshift), aligning each slice with a time offset of zero. The skull was removed from Echo 1 of the first scan (TE, 14.6 ms) and used to create a brain mask (3dSkullStrip and 3dAutomask), as this echo contained the most signal. The first Echo 2 TR from the first scan (TE, 35.79 ms) was used as a base for motion correction and registration with the T1 structural scan (3dbucket).

After completing the standard preprocessing, the data were processed using Tedana (Kundu et al., 2012, 2013; DuPre et al., 2019, 2021) to denoise the multiecho scans (version 0.0.12, using default options). In brief, the optimal combination of the three echoes was calculated, and the echoes were combined to form a single, optimally weighted time series (T2smap.py, included in the tedana package). PCA was applied, and thermal noise was removed using the tedana_orig method. Subsequently, ICA was performed, and the resulting components were classified as signal and noise based on the known properties of the T2* signal decay of the BOLD signal versus noise. Components classified as noise were discarded, and the remaining components were recombined to construct the optimally combined, denoised time series. The tedana optimally combined and denoised output was then scaled to percent signal change.

#### Experiment 2 (Dartmouth)

Multiecho data processing was implemented based on the multiecho preprocessing pipeline from afni_proc.py in AFNI (Reynolds et al., 2024). Signal outliers in the data were attenuated (3dDespike; Jo et al., 2013). After despiking, motion correction was calculated based on the second echo, and these alignment parameters were applied to all runs. We then optimally combined and denoised the data using multiecho ICA (ME-ICA) denoising (tedana.py, version 24.0.0; Kundu et al., 2012, 2013; DuPre et al., 2019, 2021) described above, with the Kundu decision tree for PCA/ICA component classification.

For task fMRI data, following tedana denoising, we applied a 3 mm Gaussian kernel (3dBlurInMask) in the volume. The time series were then normalized to percent signal change and submitted to 3dDeconvolve (see below). Deconvolved data were then mapped to each participant's surface (3dVol2Surf)

For resting-state fMRI data, we applied additional preprocessing steps to the data after tedana denoising. Specifically, we used 3dTProject to remove motion, global signal (GSR), and apply a bandpass filter (0.008–0.11 Hz). To calculate the global signal, we extracted the average activity from a whole-brain mask (gray matter, white matter, and CSF). We applied global signal regression based on evidence showing its effectiveness in removing residual effects of motion and physiological noise (Power et al., 2012, 2017, 2018). Although GSR can induce spurious negative correlations (Saad et al., 2012), our primary interest was differentiating patterns of positive connectivity. We therefore prioritized the beneficial effects of GSR for denoising because of our limited data quantity.

Following denoising, we mapped the resting-state data to the individual participant surfaces (3dVol2Surf) and smoothed them with a 3 mm Gaussian kernel (SurfSmooth).

### FMRI data analysis

#### Task deconvolution and region of interest definition—Edinburgh

The deconvolution procedure for these data is described in the earlier publication (Scrivener et al., 2025). Below we briefly describe the procedure for the reader's convenience.

##### Static scene perception

To localize the scene perception areas (OPA, PPA, MPA, and SPA), we implemented a generalized linear model (3dREMLfit; Olszowy et al., 2019) for the scene/face localizer scans. We modeled each category's activation using a block design convolved with a 16 s GAM basis function (GAM: 8.6, 0.547, 16, 3dDeconvolve). The final generalized linear model included these two task regressors. We included six parameters of motion as regressors of no interest. During regression, we estimated the temporal autocorrelation of each voxel's time series using residual maximum likelihood (REML) estimation to find the best-fit ARMA(1,1) model for the time series correlation matrix. We projected the resulting maps to each individual's cortical surface (3dVol2Surf) and smoothed them with a 2 mm Gaussian kernel.

Scene perception areas (OPA, PPA, MPA, and SPA) were defined for each participant by contrasting activity from scene versus face blocks [*t* stat > 2.79 (*p* < 0.005); surface cluster size (*k*) > 20]. SPA was defined as the peak in selectivity near the dorsal extent of the parieto-occipital sulcus and posterior intraparietal gyrus [as described in prior work (Kennedy et al., 2024; Yoon et al., 2025)].

##### Static scene memory

To localize the place memory areas (LPMA, VPMA, MPMA, and SPMA), we implemented a generalized linear model (3dREMLfit; Olszowy et al., 2019) with each imagery category's activation modeled using a BLOCK basis function [BLOCK(9.5,1), 3dDeconvolve] aligned to the onset of the imagery period. The final generalized linear model included these two task regressors. We included six parameters of motion as regressors of no interest. During regression, we estimated the temporal autocorrelation of each voxel's time series using REML estimation to find the best-fit ARMA(1,1) model for the time series correlation matrix. We projected the resulting maps to each individual's surface (3dVol2Surf) and smoothed them with a 2 mm Gaussian kernel.

We defined the place memory areas for each participant by contrasting activity from place versus people imagery trials [*t* stat > 2.79 (*p* < 0.005); surface cluster size >20]. In many subjects, this imagery contrast revealed a large cluster in the superior parietal cortex with multiple peaks of selectivity. We considered the most posterior of these peaks to be the SPMA region, and we defined SPMA by separating the cluster into its distinct peaks based on the topography of the *t* statistic. These subdivisions were made by two authors independently (R.N.T. and D.P.), and any disparity in the definition was resolved by a third author (A.S.). Importantly, because we considered the posterior cluster SPMA, subdividing this larger memory cluster into its subdivisions would make identifying a distinction between SPA and SPMA less likely.

#### Task deconvolution and region of interest definition—Dartmouth

##### Dynamic scene perception

To localize the scene perception areas (OPA, PPA, MPA, and SPA), we implemented a generalized linear model (3dREMLfit; Olszowy et al., 2019) for the multicategory localizer scans. We modeled each category's (scene, face, object, body) activation using a block design convolved with a 12 s GAM basis function (GAM: 8.6, 0.547, 12, 3dDeconvolve). The final generalized linear model included these four task regressors. We included six parameters of motion as regressors of no interest. During regression, we estimated the temporal autocorrelation of each voxel's time series using REML estimation to find the best-fit ARMA(1,1) model for the time series correlation matrix. We projected the resulting maps to each individual's surface (3dVol2Surf). Because we applied smoothing to these data before deconvolution, no further smoothing was applied.

Scene perception areas (OPA, PPA, MPA, and SPA) were defined for each participant by contrasting activity from scene versus face blocks [*t* stat > 2.79 (*p* < 0.005); surface cluster size >20]. SPA was defined as the peak in selectivity near the dorsal extent of the parieto-occipital sulcus and posterior intraparietal gyrus (Kennedy et al., 2024; Yoon et al., 2025).

##### Dynamic scene memory

To localize the place memory areas (LPMA, VPMA, MPMA, and SPMA), we implemented a generalized linear model (3dREMLfit; Olszowy et al., 2019) with a regressor for each simulation condition (house, photograph, conversing, dancing) modeled with a GAM basis function (GAM: 8.6, 0.547, 8, 3dDeconvolve) aligned to the onset of the imagery period. The final generalized linear model included these four task regressors. We included six parameters of motion as regressors of no interest. During regression, we estimated the temporal autocorrelation of each voxel's time series using REML estimation to find the best-fit ARMA(1,1) model for the time series correlation matrix. We projected the resulting maps to each individual's surface (3dVol2Surf). Because we applied smoothing to these data before deconvolution, no further smoothing was applied.

We defined the place memory areas for each participant by contrasting activity from place versus all other category simulation trials [*t* stat > 2.79 (*p* < 0.005); surface cluster size >20]. In many subjects, this imagery contrast revealed a large cluster in the superior parietal cortex with multiple peaks in selectivity. We considered the most posterior of these peaks to be the SPMA region, and we defined SPMA by separating the cluster into its distinct peaks based on the topography of the *t* statistic. These subdivisions were made by two authors independently (R.N.T. and D.P.), and any disparity in the definition was resolved by a third author (A.S.). Importantly, because we considered the posterior cluster SPMA, subdividing this larger memory cluster into its subdivisions would make identifying a distinction between SPA and SPMA less likely.

#### Topographic shift analysis

For both datasets, we analyzed the topographic shift between the scene perception and memory clusters using the approach adapted from prior work (Steel et al., 2021). After defining individual regions of interest, we quantified the anterior displacement of perception and memory areas by calculating the center of mass for the paired scene perception/place memory-selective area on each surface. We then compared the distance between the center of mass for perception and memory in the *y*-dimension (posterior–anterior) using a linear mixed-effects model with Task (Perception, Memory) and Hemisphere (LH/RH) as factors and participant as a random effect for each surface separately. We used linear mixed-effects modeling to account more effectively for participants in whom we could only identify regions in one hemisphere. Because there was no significant interaction between Task and Hemisphere (all *p*s > 0.10), we present the data collapsed across hemispheres.

We calculated the angular consistency of the shift for each region using a Rayleigh's test. We transformed the coordinates from Cartesian to polar; for this analysis, we only considered the principal orientation of the superior parietal surface (*Y* and *Z*).

To examine whether the proportion of shared vertices differed across surfaces, we compared the overlap between the category-selective perception and memory areas on each surface. We calculated the Jaccard index (proportion of shared vertices between the perception and memory areas as a function of total vertices) and compared them using a linear mixed-effects model with Surface (Medial, Ventral, Lateral, Superior parietal), Hemisphere (LH/RH) as fixed effects, and participant as a random effect.

#### Exploratory group analysis

To quantify the overall pattern of brain activity evoked by perception and memory of scenes, we performed group analyses for the experiments collected at Edinburgh and Dartmouth. We conducted four separate one-sample *t* tests for the relevant contrast maps for perception (Edinburgh, Scene v Face images; Dartmouth, Scene v Face videos) and memory (Edinburgh, Place v People imagery; Dartmouth, House v mean across social conditions) tasks from both experiments. We then qualitatively compared the location of the significant areas (*t* > 3.3; *p* < 0.008; *k* > 40) between the perception and imagery tasks in each dataset. Based on the importance of retinotopic coding to visual scene analysis, to aid interpretation, we compared the group activation maps to the known retinotopic maps in the superior parietal cortex using the Wang atlas (Wang et al., 2015).

#### Resting-state functional connectivity analyses

To investigate how SPA fits into the broader organization of the cerebral cortex, we performed two different functional connectivity analyses. To ensure that all regions were equally represented and not biased by region size, we constrained all regions of interest to the 300 vertices closest to the center of mass of the original area defined above. All original regions had >300 vertices.

##### Connectivity among the scene perception and place memory areas

To examine how the superior parietal scene perception and memory areas (SPA v SPMA) were functionally related to the originally described scene perception and memory areas, we compared the pairwise correlation of resting-state activity among all regions (perception areas, OPA, PPA, MPA, SPA; memory areas, LPMA, VPMA, MPMA, SPMA). We extracted the resting-state fMRI time series from the 300 vertex-constrained center-of-mass regions of interest. Next, to isolate the unique variance associated with each pairwise connection (Smith et al., 2013), we performed a partial correlation among all region pairs (partialling out the variance associated with other regions). We then compared the affiliation between SPA and SPMA with the perception and memory areas using a linear mixed-effects model with Hemisphere (LH/RH), Superior parietal region (SPA, SPMA), Surface (Lateral, Medial, Ventral), and the task used to define the regions (Task: Perception, Memory) as factors and participant as a random effect. We observed no effect or interaction with the hemisphere, so data are presented averaged across hemispheres.

##### Connectivity to other cortical areas

As an exploratory analysis, we used whole-brain seed-based connectivity to investigate how SPA connects with the rest of the brain. We extracted resting-state fMRI time series from the constrained SPA and SPMA ROIs and calculated, separately for each seed and hemisphere, the correlation between the seed time series and the time series at every cortical vertex. To aggregate data across participants, we created binary masks of the top 10% most connected vertices to the superior parietal perception and memory regions. This method of percentile-based thresholding has been used by prior resting-state studies to derive individualized connectivity maps (Dickie et al., 2018). We then averaged these binary masks together to derive a whole-brain probability map of SPA and SPMA connections for all participants. We conducted this analysis for each hemisphere separately.

## Results

### The posterior–anterior perception–memory motif extends to the superior parietal cortex

We began by asking whether scene perception and memory (visual imagery) tasks activate dissociable regions within the superior parietal cortex. In Experiment 1, we considered perception and visual memory tasks that closely matched prior work: (1) perception of static scene images compared with face images (Methods: Static scene perception) and (2) visual imagery of familiar places compared with visual imagery of familiar people (Methods: Static scene memory). In Experiment 2, we considered multiple social demands during both perception and recall trials: (1) perception of videos of panning scenes versus videos of children's faces (Dynamic scene perception) and (2) visual recall of familiar places compared with dynamic social simulation (Dynamic scene memory; see Materials and Methods).

For each participant, we defined regions of interest for SPA and SPMA in each hemisphere. We also defined regions of interest for the known scene perception areas and place memory areas on the medial, ventral, and lateral surfaces. Consistent with previous work, we quantified the shift in the center of mass from scene perception to place memory in each participant (in millimeters on the cortical surface), consistency of the shift direction, and the overlap between the scene perception and memory areas (Steel et al., 2021, 2025). For both scene perception and memory recall, ROIs were drawn using a vertexwise threshold of *t* > 2.79 (*p* < 0.005) and a cluster size of 20 connected surface vertices. Notably, we considered SPMA contingent on observing SPA in a participant.

**Figure 2.**
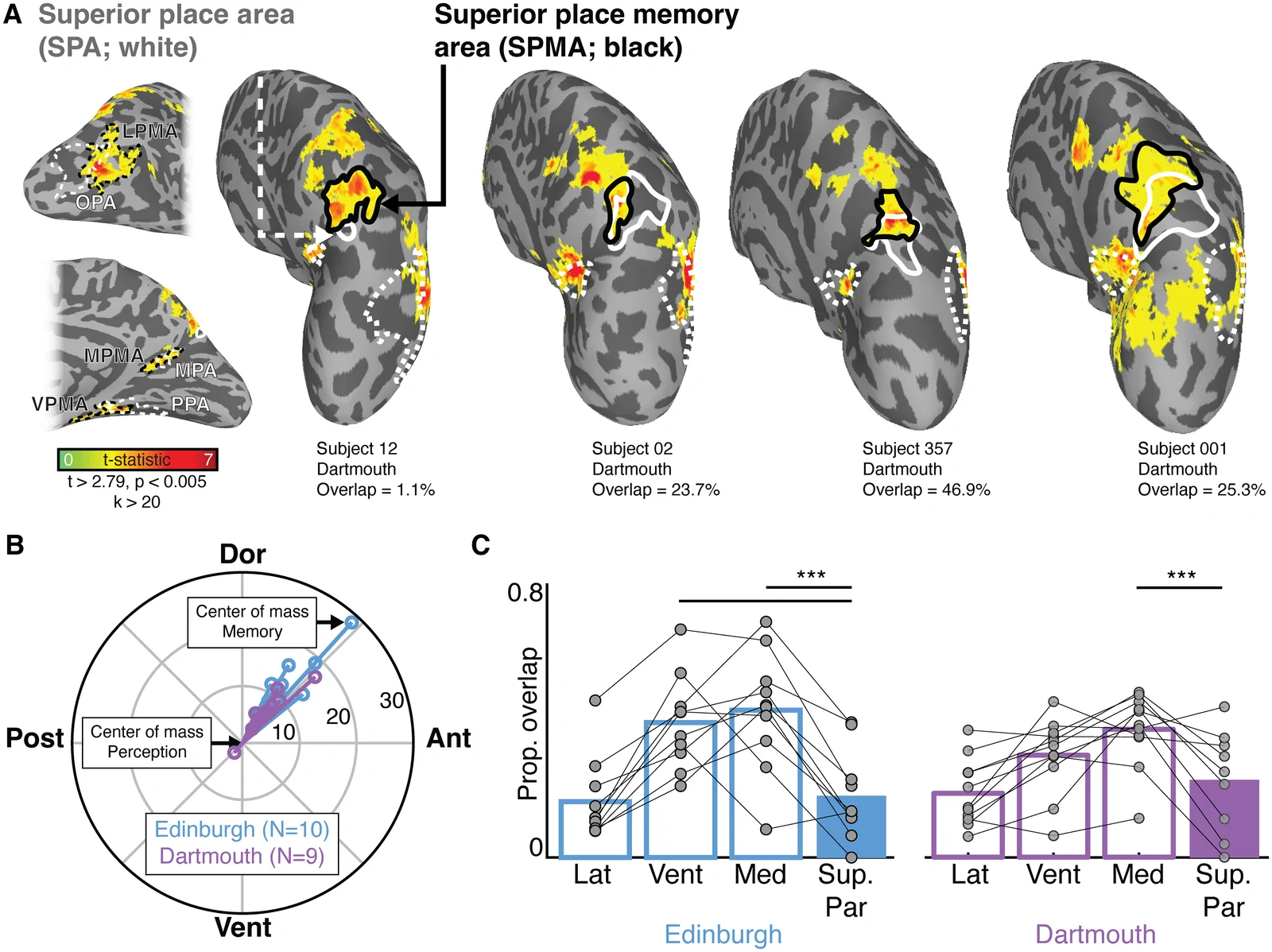
Anterior shift of scene memory versus perception activity in the superior parietal cortex. ***A***. Example scene perception (white outline) and memory area (black outline) in the superior parietal cortex in four participants. Memory activity for scene versus social imagery conditions is shown in hot colors. Lateral and medial surfaces are shown for subject 12 showing typical locations of the scene perception (white dotted lines) and place memory areas (black dotted lines). The display threshold (*t* > 2.79) was used to define the regions of interest for subsequent analyses. ***B***. Consistent anterior and dorsal shift of place memory area versus perception area in the superior parietal cortex. Perception and memory areas were drawn in individual participants in both experiments based on a vertexwise threshold (*t* > 2.79, *p* < 0.005). The center of the polar plot depicts the center of mass of the perceptual area; lines depict the shift from perception to memory area for each participant in millimeters. ***C***. Superior parietal perception and memory clusters have limited overlap compared with medial and ventral surfaces. Data points show the Jaccard index (proportion of overlapping vertices as a function of total vertices) for perception and memory areas on each surface for each participant. Data in ***B*** and ***C*** are averaged across hemispheres.

We observed a consistent topographic shift of place memory compared with scene perception in the superior parietal cortex. Observing individual participants' activation revealed a striking pattern: SPA formed the posterior border of a large cluster of scene memory activation (four example participants from Experiment 2 are depicted in Fig. 2*A*). Across participants, this large cluster of memory activity had up to four distinct peaks anterior and dorsal to SPA, spanning superior parietal lobule and the marginal ramus of the cingulate.

Because the number and distribution of local maxima within the superior parietal cortex during the memory task were idiosyncratic across participants, we defined SPMA by separating the cluster into its distinct peaks based on the topography of the *t* statistic maps (tracing local minima between cluster peaks). As a conservative definition of SPA's paired region, we considered the most posterior peak to be the SPMA. That is, we selected the peak most likely to minimize the anterior shift. Two authors independently (R.N.T. and D.P.) made these definitions, and any disparity in their definition of SPMA was resolved by a third author (A.S.). Importantly, because we considered the most posterior cluster SPMA, subdividing this larger memory cluster into its subdivisions would make identifying a distinction between SPA and SPMA less likely.

Many participants also had a peak of scene memory activity at the inflection point of the marginal ramus of the cingulate, consistent with the location of a region called “Places-2” in our prior work (Silson et al., 2019b; Scrivener et al., 2025; Scrivener and Silson, 2025). We considered this to be an independent area, and therefore we did not label it as SPMA in any participant. Because this paper focuses on the topographic adjacency of perception and memory-related activity, defining this isolated memory cluster in individuals falls outside the scope. Future studies defining this marginal ramus scene memory cluster within individuals could provide further insight into the role of the superior parietal cortex in memory-related processes.

Using this approach, we successfully localized SPA and SPMA bilaterally in most participants. In Experiment 1, we localized SPA in 10/12 participants; all participants with SPA had an associated memory area (10/10). In Experiment 2, we localized SPA in 11/12 participants; among these 11 participants, we observed SPMA in 9/11 participants bilaterally. Note that the smaller number of participants with SPMA in Experiment 2 could be due to the complexity of the memory task and the smaller number of scene memory trials in this task. This suggests a robust response during scene memory recall within the superior parietal cortex, complementing the description of SPA in perceptual tasks (Kennedy et al., 2024; Yoon et al., 2025).

Across both experiments, all but one participant showed an anterior shift of perception versus memory activation in the superior parietal cortex (Fig. 2*B*; Experiment 1, 10/10; Experiment 2, 8/9; across experiments = 94.73%). At the group level, this anterior shift was significant in both experiments, with a mean anterior-posterior shift of 7.73 (range, 0.75–23.1) and 4.48 (−1.45 to12.71) mm in Experiments 1 and 2, respectively (linear mixed-effects model; main effect of task, Experiment 1, *F*_(1,36)_ = 11.58; *p* = 0.0016; *η*^2^ = 0.243; *t*_(9)_ = 4.86; *p* = 0.00090; *d* = 1.378; Experiment 2, *F*_(1,36)_ = 10.148; *p* = 0.0029; *η*^2^ = 0.215; *t*_(8)_ = 3.41; *p* = 0.009; *d* = 1.136). There was no interaction between hemisphere and task in either experiment (LME, Hemisphere × Task, Experiment 1, *F*_(1,36)_ = 0.009; *p* = 0.92; *η*^2^ < 0.01; Experiment 2, *F*_(1,36)_ = 0.016; *p* = 0.89; *η*^2^ < 0.01). This shift was consistently anterior and dorsal, which we confirmed based on a Rayleigh's test of uniformity (Experiment 1, *z* = 7.934; *p* < 0.0001; Experiment 2, *z* = 6.28; *p* = 0.00061).

In addition to the superior parietal cortex, we confirmed that these new samples of participants showed the anterior shift of memory versus perception across the other cortical surfaces (Steel et al., 2021, 2025). All regions of interest were present in at least one hemisphere in 10/12 participants in Experiment 1 and 11/12 participants in Experiment 2. Consistent with prior reports, we observed the anterior shift of memory compared with perception activity for scenes in both Experiments on the lateral and ventral surfaces (linear mixed-effects model, main effect of task and follow-up *t* tests: Experiment 1, Lateral, *F*_(1,36)_ = 130.63; *p* < 0.0001; *η*^2^ = 0.789; *t*_(9)_ = 11.75; *p* < 0.0001; *d* = 3.72; Ventral, *F*_(1,36)_ = 33.55; *p* < 0.0001; *η*^2^ = 0.482; *t*_(9)_ = 7.11; *p* < 0.0001; *d* = 2.25; Experiment 2, Lateral, *F*_(1,41)_ = 87.85; *p* < 0.0001; *η*^2^ = 0.682; *t*_(10)_ = 12.13; *p* < 0.0001; *d* = 3.66; Ventral, *F*_(1,41)_ = 16.67; *p* = 0.0002; *η*^2^ = 0.289; *t*_(10)_ = 4.17; *p* = 0.0019; *d* = 1.26). On the medial surface, we observed no anterior shift in Experiment 1 and a tendency for a posterior shift in Experiment 2 (Experiment 1, *F*_(1,36)_ = 1.26; *p* = 0.27; *η*^2^ = 0.034; *t*_(9)_ = 0.75; *p* = 0.47; *d* = 0.23; Experiment 2, *F*_(1,42)_ = 6.53; *p* = 0.01; *η*^2^ = 0.135; *t*_(10)_ = −1.08; *p* = 0.304; *d* = 0.33). This further strengthens the evidence for an anterior shift on the lateral and ventral surfaces (Steel et al., 2025).

All of the place memory areas tend to be anteriorly shifted compared with their perceptual counterparts. However, the degree of overlap between these areas differs by cortical surface, with the lateral, ventral, and medial surfaces ordered least to greatest (Steel et al., 2021). How would SPA and paired memory area fit within this range? Intriguingly, the proportion of overlap between perception and memory activation in the superior parietal cortex was similar to the OPA and LPMA on the lateral surface (Fig. 2*C*). When we compared the Jaccard index (proportion of overlapping vertices vs total) across all surfaces (medial, ventral, lateral, superior parietal), we found the proportion of overlapping vertices was significantly lower in the superior parietal versus the medial and ventral surfaces in Experiment 1 (LME, Main effect of Surface, Experiment 1, *F*_(3,73)_ = 13.144; *p* < 0.0001; *η*^2^ = 0.354; follow-up *t* tests, Experiment 1, Sup. Par. vs Ventral *t*_(9)_ = 3.58; *p* = 0.0059; *d* = 1.13; Sup. Par. vs Medial *t*_(9)_ = 5.72; *p* = 0.00029; *d* = 1.80; Sup. Par. vs Lateral *t*_(9)_ = −0.32; *p* = 0.75; *d* = 0.10; Lateral vs Ventral *t*_(9)_ = −6.85; *p* < 0.0001; *d* = 2.17; Lateral vs Medial *t*_(9)_ = −5.69; *p* = 0.00030;, *d* = 1.80; Ventral vs Medial *t*_(9)_ = −0.63; *p* = 0.55; *d* = 0.2). In Experiment 2, we found significantly lower overlap between the place memory and scene perception areas in the superior parietal cortex compared with the medial surface and a numerically lower overlap compared with the ventral surface (although this was not statistically significant; LME, Main effect of Surface, Experiment 2, *F*_(3,73)_ = 4.467; *p* = 0.006; *η*^2^ = 0.153; follow-up *t* tests, Sup. Par. vs Ventral, *t*_(8)_ = 1.902; *p* = 0.094; *d* = 0.63; Sup. Par. vs Medial, *t*_(8)_ = 3.88; *p* = 0.005; *d* = 1.293; Sup. Par. vs Lateral, *t*_(8)_ = −0.603; *p* = 0.563 *d* = 0.20; Lateral vs Ventral, *t*_(10)_ = −3.56; *p* = 0.005; *d* = 1.07; Lateral vs Medial, *t*_(10)_ = −4.478; *p* = 0.001; *d* = 1.35; Ventral vs Medial, *t*_(10)_ = −2.310; *p* = 0.044; *d* = 0.70).

### Scene perception and memory activity border parietal retinotopic maps

**Figure 3.**
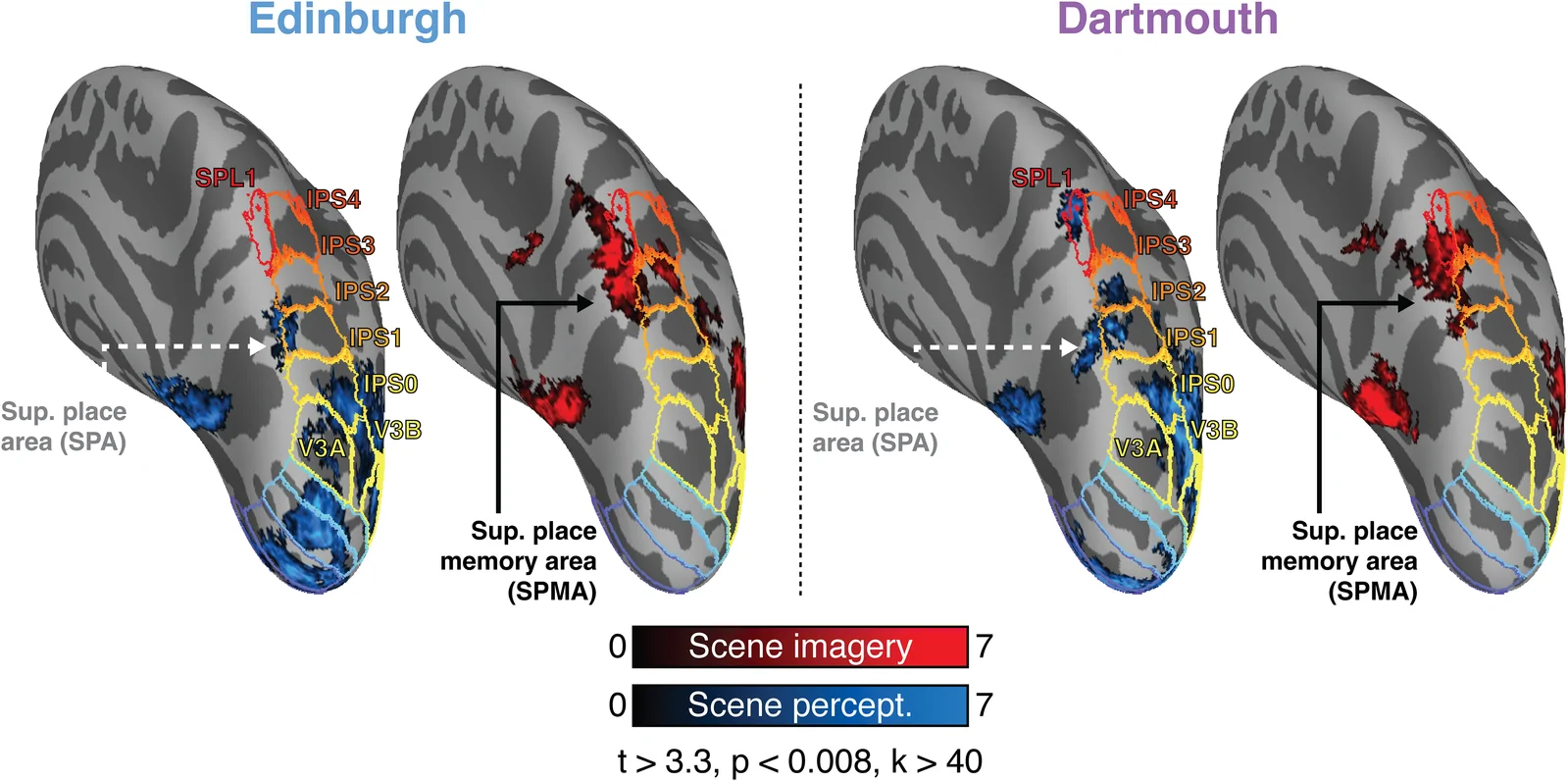
Scene perception and place memory activity occur in distinct locations in the superior parietal cortex. We conducted a group analysis of the scene perception (blue) and memory (red) conditions for each experiment and compared these results to probable locations of known retinotopic maps in the intraparietal sulcus [defined using Wang et al. (2015); group analysis thresholded at *t* > 3.3; *p* < 0.008; *k* > 40]. In both experiments, scene perception activity occurred within the retinotopic map IPS1, while scene memory activity straddled the medial boundary of maps IPS2, IPS3, and SPL1. In both experiments, we observed additional scene memory activity at the inflection point of the marginal ramus of the cingulate, consistent with the region “Places-2” (Silson et al., 2019b; Scrivener et al., 2025; Scrivener and Silson, 2025).

Analysis of individual participants' activity provided strong support for the distinction between the perception and memory areas in the superior parietal cortex. To highlight the consistency across experiments and situate these findings with prior literature, we performed an exploratory group analysis of the scene perception and memory tasks separately for both experiments (Fig. 3). All participants (including those in whom we could not localize SPA or SPMA individually) were used in this group analysis. We compared the group activation maps to the known retinotopic maps from the Wang atlas (Wang et al., 2015) based on the importance of retinotopic coding in perception and memory (Silson et al., 2019b; Breedlove et al., 2020; Groen et al., 2021; Favila et al., 2022; Angeli et al., 2024; Steel et al., 2024a,b; Scrivener and Silson, 2025).

Our group analysis revealed highly consistent areas of activation across experiments, with scene memory activity anteriorly shifted relative to perception. For scene-selective perceptual activity, we observed a cluster in the medial portion of IPS1 at the dorsal extent of the parieto-occipital sulcus, consistent with the location of SPA (Kennedy et al., 2024; Yoon et al., 2025). At our threshold (*t* > 3.3; *p* < 0.008; cluster size >40), this cluster minimally overlapped with IPS2. OPA overlapped with V3B, LO1, LO2, and IPS0 (Silson et al., 2016a; Scrivener et al., 2024) and was separated from SPA by nonsignificant vertices in the lateral portion of IPS1. In Experiment 2, in addition to the group SPA cluster, we observed another patch of scene-selective perceptual activity in the superior parietal cortex within the anterior portion of the retinotopic map SPL1. Thus, despite Experiments 1 and 2 using static versus dynamic stimuli, we observed common patterns of scene-selective activity in superior parietal cortex, suggesting robust localization of SPA regardless of the stimulus type.

Like the scene perception activation, scene memory activity was consistent across experiments and linked to IPS retinotopic maps. We observed a cluster of scene memory activity anterior to IPS1, spanning the medial portion of IPS2, IPS3, and the posterior portion of SPL1. Importantly, this group SPMA memory cluster straddled the medial edge of these maps, partially overlapping with these maps and extending further medially into the precuneus on the medial wall. Notably, at our threshold (*t* > 3.3), in Experiment 2, the group SPMA terminated posterior to the second scene perception cluster within anterior SPL1, indicating a possible distinction in perception and memory activity within the SPL1 retinotopic map.

In addition to this SPMA cluster, we observed another patch of memory-related activity anterior and medial to SPMA. This cluster, likely corresponding to the Places-2 memory cluster (Silson et al., 2019b; Scrivener and Silson, 2025; Scrivener et al., 2025) or anterior precuneus (Zhang et al., 2025), fell at the inflection point of the marginal ramus of the cingulate on the medial wall and did not overlap with any retinotopic maps. Thus, the medial parietal cortex appears to be tiled by three place memory-selective regions: a ventral region within the parieto-occipital sulcus (MPMA/Places-1), a dorsal region located within the superior parietal lobule (SPMA), and an anterior region at the inflection point within the cingulate ramus.

### Place memory areas maintain selectivity during visual perception

Our prior analysis shows that recalling personally familiar places activates brain areas anterior to the scene perception areas. A related question is whether the place memory areas retain their selectivity when perceiving visual scenes. To address this question, we extracted the activity estimates (*β* values) from the place memory areas [including SPMA in the dynamic localizer task collected during Experiment 2 (Dartmouth)]. We focused on the Dartmouth dataset because this experiment included rest blocks (12 s), allowing us to assess the activation during perception versus a fixation baseline. To equate SNR and signal homogeneity across the regions, we constrained these regions to the 300 vertices closest to each region's center of mass.

**Figure 4.**
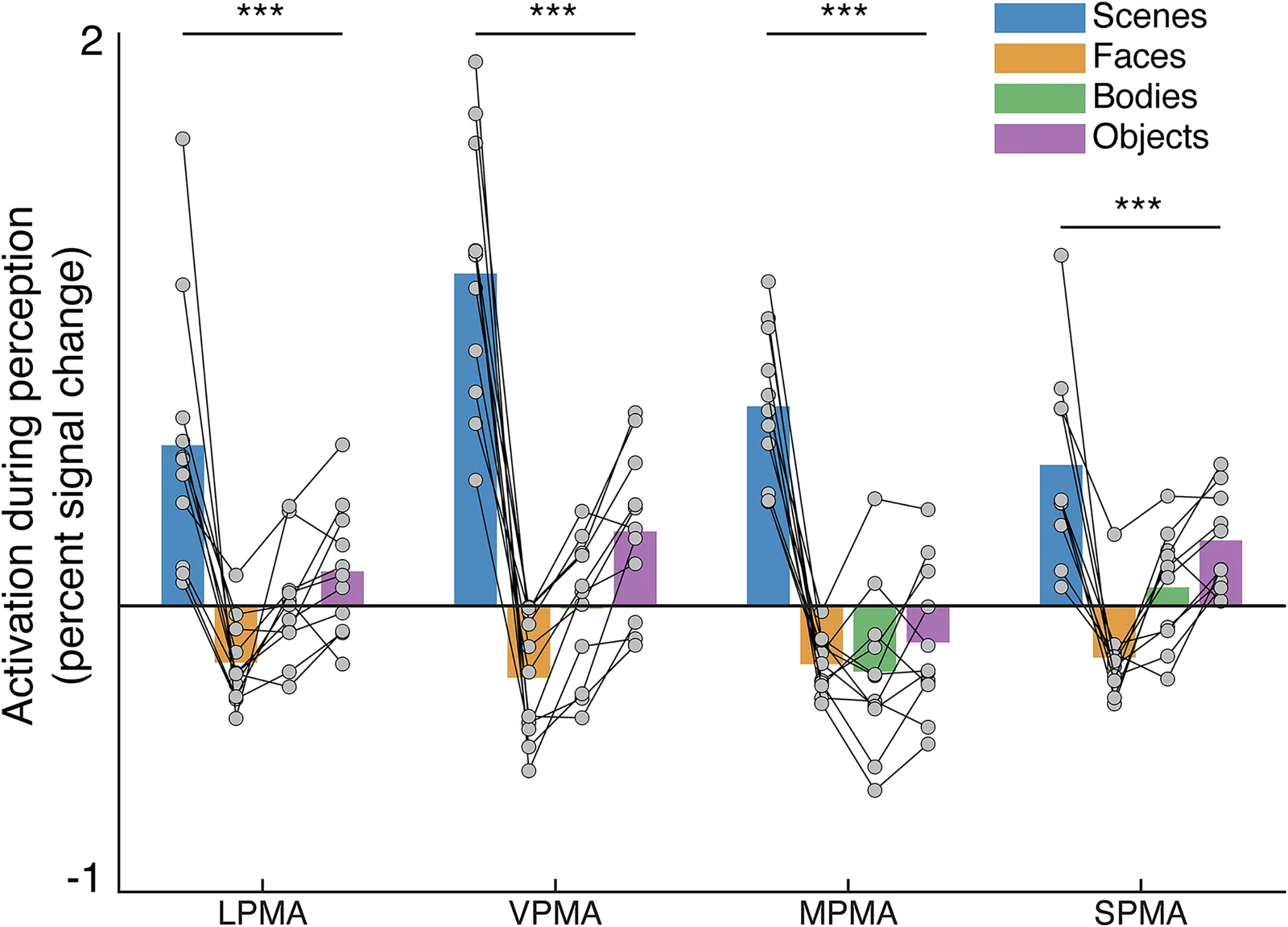
The place memory areas activate in a relatively selective manner to visually presented scenes. We extracted activation (*β* values) during the dynamic perception task from the place memory areas in participants from Study 2 (Dartmouth). All place memory areas showed greater activity during scene perception compared with other categories (faces and bodies), although SPMA was not significantly different from objects. This is consistent with the general preference for spatial information, even in the absence of memory recall demands.

We found that the place memory areas activated in a relatively selective manner during scene perception, consistent with the partial overlap between the scene perception and place memory areas (Fig. 4). Across all ROIs, we observed a Main effect of Category (LPMA. *F*_(3,76)_ = 12.96; *p* < 0.001; *η*^2^ = 0.334; VPMA, *F*_(3,76)_ = 33.973; *p* < 0.001; *η*^2^ = 0.573; MPMA, *F*_(3,76)_ = 20.708; *p* < 0.001; *η*^2^ = 0.437; SPMA, *F*_(3,68)_ = 8.931; *p* < 0.001; *η*^2^ = 0.283). Critically, viewing scenes evoked greater activity compared with all other categories in LPMA, VPMA, and MPMA (*t*s > 3.41; *p*s < 0.04). The notable exception was SPMA, where we observed greater activity during scene perception compared with faces (*t*_(8)_ = 5.395; *p* = 0.003; *d* = 1.70) and bodies (*t*_(8)_ = 3.986; *p* = 0.019; *d* = 1.26), but not objects (*t*_(8)_ = 1.934; *p* = 0.511; *d* = 0.612). This suggests that, although these regions are preferentially active during memory tasks, they retain their selectivity when perceiving dynamic visual scenes.

### Connectivity dissociates superior parietal scene perception and memory areas

Analyzing coactivation of brain areas can provide insight into their function, because coactivation patterns at rest often reflect contributions to similar cognitive/functional processes (Biswal et al., 1995). When watching a video meant to convey naturalistic scene understanding, the scene perception and memory areas on the lateral, ventral, and medial surfaces form distinct functional networks (Steel et al., 2021). Here, we asked whether this distinction between the scene perception and memory areas was also true during rest and whether the scene perception and memory areas in the superior parietal cortex would preferentially associate with regions defined in the same task.

We hypothesized that activity of SPA would be more correlated with the scene perception areas (OPA, PPA, and MPA), while SPMA would be more correlated with the memory areas (LPMA, VPMA, and MPMA). We tested this hypothesis in resting-state fMRI data collected in Experiment 2 (Dartmouth) participants in whom we could define all perception and memory areas (*N* = 9). This sample size was sufficient to detect a large effect (Cohen's *d* > 1.2; 80% power; *α* = 0.05), which is consistent with the functional connectivity between scene perception and place memory areas detected in our prior study (Cohen's *d*s > 2.39; Steel et al., 2021). As above, to equate SNR and signal homogeneity across the regions, we constrained these regions to the 300 vertices closest to each region's center of mass. We extracted the resting-state fMRI time series from these constrained ROIs and calculated the partial correlation among all region pairs (while partialling out variance shared with all of the regions). After constraining to the 300 vertices around the centers of mass, ROIs minimally overlapped (all ROIs < 50% overlap) and results did not differ if only unique vertices were included.

**Figure 5.**
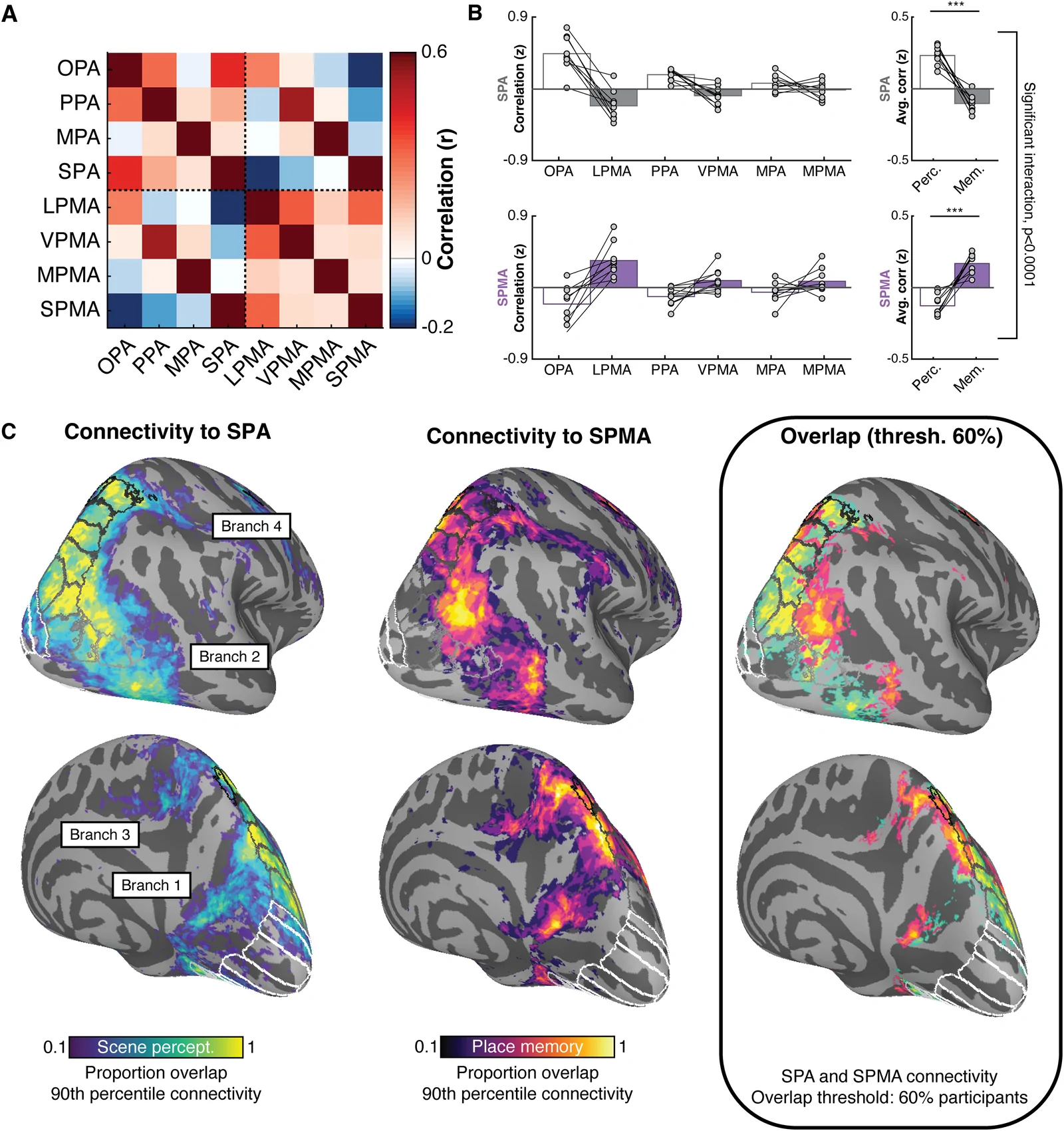
Functional connectivity during rest dissociates perception and memory areas in the superior parietal cortex. ***A***. We calculated the pairwise partial correlation (Smith et al., 2013) between scene perception areas (OPA, PPA, MPA, SPA) and place memory areas (LPMA, VPMA, MPMA, SPMA), providing an estimate of the unique variance at each connection. ***B***. Differential connectivity between SPA and SPMA with the other scene perception and place memory areas. SPA was more connected with other scene perception areas, while SPMA was more connected with the place memory areas. ***C***. Parallel branching connectivity streams within the superior parietal cortex emanating from SPA (left) and SPMA (middle). In each participant, we calculated whole-brain connectivity of SPA and SPMA and then created binarized masks of the 90th percentile of connected vertices. We aggregated these masks across participants to create a probability map of SPA and SPMA connectivity. This analysis revealed parallel streams of connectivity spanning the superior parietal cortex and intraparietal sulcus. Connectivity from SPA largely follows IPS, with branches emanating (1) posterior ventrally (toward OPA/LPMA), (2) posterior medially (toward MPA/MPMA), (3) dorsal medially (toward marginal ramus of the cingulate, Places-2), and (4) dorsal laterally (toward parietal area PF). Connectivity from SPMA borders these connectivity streams on the lateral and medial sides, with peaks of connectivity falling anterior to the peaks of SPA connectivity at each branch's terminus. The right panel depicts the SPA and SPMA probability maps, showing vertices that exceeded a 60% overlap across participants.

Two principles appeared to organize the functional relationship among the perception and memory areas. The first principle was anatomical pairing: we observed high connectivity strength between anatomically paired perception and memory areas (Fig. 5*A*, secondary-diagonal). The second principle was perceptual versus mnemonic region: aside from the paired region, all other across-network connections (i.e., perception to memory area pairs) were lower than within-network connections. Thus, these regions' resting-state activity captures their association during the localizer tasks.

With respect to perception and memory areas in the superior parietal cortex, SPA and SPMA were affiliated with the other scene perception and memory areas at rest. We observed a double dissociation in the connectivity between the superior parietal perception and memory areas with the other surfaces' scene perception and memory areas (Fig. 5*B*; LME, Superior parietal area × PA/MA interaction, *F*_(1,96)_ = 135.7; *p* < 0.001; *η*^2^ = 0.586). This was driven by preferential connectivity of SPA to the other perceptual versus memory areas and vice versa for SPMA (Difference SPA v SPMA connectivity for perception vs memory areas, *t*_(8)_ = 4.165; *p* = 0.003; *d* = 1.388; Perception v memory area connectivity, SPA,: *t*_(8)_ = 8.591; *p* < 0.001; *d* = 2.864; SPMA, *t*_(8)_ = −6.715; *p* < 0.001; *d* = 2.238), although this preference was qualitatively weaker for the medial surface than the ventral and lateral surfaces. Notably, all participants showed this double dissociation. This is consistent with the hypothesis that the scene perception and place memory areas constitute dissociable functional networks, including the new regions within the superior parietal cortex.

### Distinct medial and lateral connectivity streams originate from the superior parietal perception area

Analysis of the topography of scene perception and memory activation in the superior parietal cortex showed that SPA formed the posterior boundary of a larger swath of memory-evoked activity within this region. This led us to ask how SPA and SPMA functionally connect with the rest of the cortex, particularly the superior parietal cortex anterior to SPA.

To address this question, we conducted an exploratory seed-based connectivity analysis of SPA and SPMA. We extracted resting-state fMRI time series from the individually defined SPA (*n* = 10) and SPMA regions (*n* = 9) and calculated the whole-brain correlation map with each seed (separately for each hemisphere). We then created a binary mask of the top 10% most connected vertices for each participant and aggregated these binary masks across participants to create a vertexwise probability map. Although we did not explicitly stipulate a positivity constraint, only positive connectivity values were included in the 10% connectivity mask. Using this binary mask approach (vs other group analysis approaches like *t* testing) prevents bias from outlying connectivity values within individual participants and ensures that all participants contribute equally to the group-level map.

This analysis revealed that SPA and SPMA connectivity form parallel pathways extending throughout the parietal cortex. SPA sits at the posterior confluence of four anatomically distinct branches of preferential connectivity extending into the dorsal parietal cortex. These branches are bordered on their medial and lateral sides by areas with high connectivity with SPMA. Figure 5*C* shows the proportion of overlapping participants’ connectivity maps from SPA (left), SPMA (middle), and the overlap between these maps thresholded at 60% overlapping participants (right).

Ventrally, connectivity from SPA follows two distinct branches. Branch 1 runs laterally, connecting SPA to OPA. Branch 2 runs medially, connecting SPA to MPA/MPMA via the parieto-occipital sulcus. These connectivity streams converge at SPA and form a single stream moving anterior and dorsally, through SPMA toward the superior parietal cortex. Both branches are bordered by increased connectivity with SPMA, the lateral stream beginning in LPMA, and the medial stream beginning in MPA/MPMA.

Dorsally, the connectivity stream emanating from SPA splits into two additional dorsal medial and lateral branches. In the group-average map, the bifurcation appeared within the superior aspect of the marginal ramus of the cingulate and the retinotopic map SPL1. At the bifurcation point, Branch 3 dove down the medial wall toward the inflection point of the marginal ramus of the cingulate. Branch 4 extended ventrally and laterally into the PF complex and area PFt in the lateral parietal cortex (Glasser et al., 2016). The end points of Branches 1–3 have recognized roles in navigation-related processes (Marchette et al., 2014; Persichetti and Dilks, 2018; Zhang et al., 2025), and area PF/PFt has recently been identified as a member of a larger brain network related to navigation (Zhang et al., 2025). Connectivity from SPMA followed these branches toward their termini, paralleling their medial and lateral trajectories. This positions the superior parietal cortex as an anatomical hub connecting disparate cortical areas to facilitate visually guided navigation.

In addition to these connectivity pathways in the superior parietal cortex, we observed three other loci of increased SPA and SPMA connectivity distributed in the cortex. Interestingly, these areas tended to be at the anterior edges of retinotopic maps (Wang et al., 2015), and at each location, SPMA connectivity fell at the anterior edge of the SPA connectivity cluster. We observed one patch of connectivity in the medial ventral temporal cortex, encompassing the retinotopic maps VO1 and 2 and PHC1 and 2, consistent with the location of PPA and VPMA. A second patch of connectivity was in the lateral occipitotemporal cortex, anterior to the retinotopic maps MT/hMT. Finally, we found a third patch in the lateral prefrontal cortex, anterior to the frontal eye fields. These connectivity peaks at anterior portions of the visual retinotopic maps suggest a broader linkage between areas at the boundary of classically defined visual areas.

## Discussion

Integrating sensory and mnemonic information is a core problem faced by the brain (Rademaker et al., 2019; Favila et al., 2020; Groen et al., 2021; Rust and Palmer, 2021; Steel et al., 2024b). In the context of scenes, prior work has shown that colocalization of perceptual and memory-related brain areas facilitates their interaction: immediately anterior to the lateral and ventral scene perception areas exists a memory-responsive region that represents visuospatial context currently out of view (Silson et al., 2016b, 2019a; Steel et al., 2021, 2023, 2025; Srokova et al., 2022). Here, we show that the proposed fourth scene perception area in the superior parietal cortex, SPA (Kennedy et al., 2024; Yoon et al., 2025), also exhibits this motif. Using individualized fMRI stimuli and analyses, we show that SPA sits at the posterior edge of a large swath of the cortex that responds selectively during recall of scenes (SPMA). Resting-state functional connectivity confirmed that SPA and SPMA differentially affiliate with the other scene perception and place memory areas, supporting roles in visual analysis versus representing visuospatial context, respectively. Finally, SPA sits at the intersection of two connectivity pathways with four branches: a medial pathway linking the retrosplenial cortex and dorsal precuneus and a lateral pathway linking the caudal inferior parietal lobe to the superior parietal and prefrontal cortex. These data suggest that SPA and SPMA may be uniquely positioned to coordinate mnemonic and perceptual reference frames to guide attention processes during visually guided navigation.

Two studies have recently proposed adding a fourth region (SPA) to the set of brain areas considered important for scene perception (OPA, PPA, and MPA; Kennedy et al., 2024; Yoon et al., 2025). Our data generally support the reported location and consistency of SPA. We observed SPA in the vast majority of our participants across two independent datasets, which used static (Experiment 1) and dynamic (Experiment 2) stimuli. In both datasets, this area was located at the dorsal extent of the parieto-occipital sulcus, separate from OPA. At the group level, SPA fell adjacent to the retinotopic map IPS1 in both datasets and extended anteriorly into IPS2 in the group of participants who viewed dynamic stimuli. The anatomical location and preference for dynamic versus static stimuli agree with prior reports; however, our two datasets were collected independently, so we interpret this difference across datasets with caution. Notably, prior descriptions of PIGS located this area adjacent to the retinotopic IPS3 and IPS4 (Kennedy et al., 2024), which is anterior and dorsal to our group analysis and more consistent with our memory-evoked activity. This discrepancy may be due to the more precise individualized retinotopic map definition in past work compared with our group atlas approach. Thus, while prior work investigating the neural basis of scene perception has focused on three areas (Epstein and Baker, 2019; Dilks et al., 2022), future work focused on visually guided navigation may also incorporate SPA.

Using mental imagery of scenes to isolate top–down processes, we found that SPA falls at the posterior boundary of a larger memory-responsive cluster extending through precuneus toward the marginal ramus of the cingulate. We could localize this memory-responsive region, SPMA, in the majority of participants across two studies despite different tasks and experimental parameters, which speaks to its robustness. Like the other memory-responsive regions (Steel et al., 2021, 2025), SPMA was anatomically distinct from its perceptual counterpart, with limited overlap between these functionally defined areas. Interestingly, when viewing dynamic stimuli, SPMA retained its scene selectivity versus faces, suggesting that the preference for scenes in memory extends into perception in these areas at a subthreshold level. Although this pattern may be driven by the small overlapping area between these functional regions of interest, further work should continue to resolve the functional dissociation of the perception and memory areas. The ability to localize SPA and SPMA in individual participants suggests that they are meaningful features of brain organization (Petersen et al., 2024) and opens up the possibility of studying these functional areas' specific roles in coordinating scene perception and visuospatial memory.

The distinction between perception and memory is largely consistent on the lateral, ventral, and superior parietal surfaces, while these processes tend to overlap on the medial surface. The medial surface responds strongly to mnemonic and spatial context, including familiarity with scenes and spatial knowledge (Bar and Aminoff, 2003; Sugiura et al., 2005; Epstein et al., 2007a,b; Park and Chun, 2009; Robertson et al., 2016; Persichetti and Dilks, 2018, 2019; Berens et al., 2021). The lack of a difference between the SPA and SPMA's connectivity with the medial areas further supports this similarity. One possibility is that the area within the parieto-occipital sulcus constitutes a unified perception–memory architecture rather than a topographically distinct pairing. We did not observe a consistent anterior shift from perception to memory in this region of cortex, consistent with other work that has also failed to observe this effect (Steel et al., 2025). In addition our prior work found similar responses to increasing spatial knowledge during perception and imagery in MPA and MPMA (Steel et al., 2023). A second possibility is a more complex distinction that is not well captured by the simple “anterior shift” heuristic. For example, other work has observed a perception–memory distinction on the medial surface, including distinct peaks in activity across the boundary of the parieto-occipital cortex (Silson et al., 2019a) and intermixed positive and negative retinotopic coding in this area (Scrivener and Silson, 2025). Resolving the linkage between perception and memory processes within the parieto-occipital sulcus will likely require targeted investigation of this area, including tasks designed specifically to engage this region and high-resolution fMRI to disambiguate the anterior and posterior banks of the sulcus.

What navigation processes might the scene perception and memory areas in superior parietal cortex serve? Although SPA has only recently been added to the “scene perception areas,” prior work has implicated the superior parietal cortex in navigationally relevant processes. Patients with superior parietal lesions exhibit “egocentric disorientation,” characterized by an inability to locate objects and navigational affordances relative to themselves (Aguirre and D’Esposito, 1999). In addition, fMRI studies have noted superior parietal activation during egocentric navigationally relevant tasks (Sulpizio et al., 2023), including integrating information across changes in viewpoint (Van Assche et al., 2014), evaluating paths through a scene (Persichetti and Dilks, 2018), judging relative directions between viewpoints from memory (Marchette et al., 2014; Huffman and Ekstrom, 2019), and actively navigating (Suzuki et al., 2021). One study that employed a similar memory localizer task to our Edinburgh dataset found that the superior parietal cortex appeared to represent integrated local viewpoints (Han and Epstein, 2026). In addition, two recent studies using an encoding model approach to fMRI data analysis provide additional, fine-grained insight into the superior parietal cortex's role in navigation (Lu et al., 2025; Zhang et al., 2025). One elegant study employing a navigation task in identical but visually distinct environments observed precise head direction coding uniquely in the superior parietal cortex and in RSC/MPA (Lu et al., 2025). A similar study with a large navigable environment and an elaborate set of navigation-relevant analytical regressors also observed head direction coding in the superior parietal cortex, along with a mixture of motion energy, gaze semantics, and future path (Zhang et al., 2025). Our work adds to this evidence, showing that simply visually recalling navigationally relevant information from an egocentric perspective is sufficient to activate the superior parietal cortex.

SPA sits at a junction of four distinct functional connectivity branches: two medial branches between the parieto-occipital sulcus and dorsal precuneus and two lateral branches between lateral occipital and parietal cortices. These branches were bordered by two parallel streams of connectivity to SPMA, with each SPMA stream's endpoint falling anterior to SPA's. This finding reinforces two key points. First, these results show that perception and memory processes are intimately paired throughout the cortex, and the superior parietal cortex appears to be an area in which these processes come together. Second, they further support the expanded role of superior parietal cortex in navigation function. Importantly, these branches span regions implicated in navigation-relevant processes, including allocentric map-like navigation (Marchette et al., 2014; Persichetti and Dilks, 2018; Lu et al., 2025; Naveilhan et al., 2025), perspective taking (Zhang et al., 2025), and egocentric scene perception (Pitcher et al., 2011; Persichetti and Dilks, 2016, 2019). Being situated at the intersection of these streams puts the superior parietal cortex, home to SPA and SPMA, in a unique position as an integrative hub, where representation of the current visual percept is connected with our internal representation of our location and goal to guide our attention and inform our movement.

In summary, successful navigation requires both extracting visual information from the current scene and integrating that input with stored knowledge of the broader environment. A path is only viable if you know where it leads, and a landmark is only useful if you know what it signifies. Each scene perception area extracts a spatially biased slice of the current visual scene (Silson et al., 2015, 2016a). However, that slice is only meaningful in light of context that extends beyond what is currently visible.

The architecture of the scene processing cortex is organized to meet this demand: the scene perception areas are anatomically coupled with memory-responsive partners. This organization is particularly prominent on the lateral, ventral, and superior parietal surfaces, which clearly exhibit this posterior–anterior perception–memory motif. While the shift in activity is less prominent on the medial surface, memory recall evokes activity in a larger swath of the cortex in this region, suggesting that MPA may be a visually responsive subarea within a larger territory involved in memory-related processing. Crucially, each memory area connects to holistic representations of space in the hippocampus and our goals in the prefrontal cortex. This may allow contextual information from the memory areas to be fed back to shape ongoing perceptual processing (Angeli et al., 2024; Steel et al., 2024a,b), such that what OPA “sees” as a viable path or what PPA recognizes as a meaningful landmark is jointly determined by current visual input and stored knowledge of the environment. In this sense, “visually guided navigation” is not the function of a particular brain area—it emerges from the connection between each scene area's visual computations and the context that gives them meaning.

## Synthesis

Reviewing Editor: Niko Busch, Westfalische Wilhelms-Universitat Munster Decisions are customarily a result of the Reviewing Editor and the peer reviewers coming together and discussing their recommendations until a consensus is reached. When revisions are invited, a fact-based synthesis statement explaining their decision and outlining what is needed to prepare a revision will be listed below. The following reviewer(s) agreed to reveal their identity: Andrew Persichetti, Linfeng Tony Han. Note: If this manuscript was transferred from JNeurosci and a decision was made to accept the manuscript without peer review, a synthesis may not be available.

# Review Synthesis

Both reviewers evaluated this manuscript positively and agree that it makes a clear and timely contribution to the understanding of the cortical organization of scene processing. The analyses are considered thorough, and the manuscript is well written. The suggestions from both reviewers are relatively minor in nature, consisting mainly of requests for clarification and elaboration. Reviewer #2 raises one more substantive point regarding the framing of the medial surface results and the "ubiquitous" motif claim, which should be addressed in the discussion. In the post-review consultation, both reviewers agreed that the MDS analysis (Figure 4B) is difficult to interpret as presented and should be clarified or reconsidered. Several of the points raised by Reviewer #1 may be addressable in the reply letter alone; the authors may decide whether they prefer to revise the manuscript accordingly.

# Reviewer 1

The authors ran two separate experiments to demonstrate that a visual scene-selective region in the superior parietal lobe (SPL) follows the same perception-memory motif as the other cortical scene regions (i.e., OPA, PPA, MPA). Specifically, the authors showed participants static (i.e., pictures) scenes and faces (experiment 1), and dynamic (i.e., short videos) scenes, faces, bodies, and objects (experiment 2). They defined the perceptual scene-selective region in SPL (and the other cortical scene regions) using the contrast, scenes > than non-scene stimuli. In addition to these visual stimuli, participants were also asked to imagine the different categories and the authors again used the same contrast to look for the memory-specific scene region in SPL (and the other cortical scene regions). In both datasets the authors found that predicted perception memory motif in SPL - i.e., recalling scenes evoked responses immediately anterior and dorsal to the region responding to the visually presented scene stimuli. In addition to the localization of the memory-responsive SPL scene region (SPL-mem), the authors, include an analysis of how it relates to retinotopic maps in the parietal lobe and a resting-state analysis to show how the SPL scene region is connected to the rest of the cortical scene network.

The analyses are reasonable and thorough, and the manuscript is clearly written. In fact, I find very little to critique in this manuscript. Below are some minor suggestions that the authors might choose to address in the final draft.

1. SPL localization in each hemisphere. The criteria you used for SPL definition was not clear to me. You mention that you found SPL bilaterally in almost every participant. Did you exclude participants if you found SPL in only one hemisphere or did you include them since you found no differences between hemispheres anyway? You mention that the other "regions of interest were present in at least one hemisphere". Did you use different criteria for the SPL and the other ROIs? A little clarification in the revised manuscript could be helpful.

2. Connectivity from the SPL-mem? The resting-state functional connectivity analysis uses the scene perception region in SPL as a seed. Why not do the same for the SPL-mem?

3. How does SPL-mem respond to visually presented scenes? The only shortcoming of this paper is that it does not include an independent set of conditions to probe the selectivity of the functionally defined ROIs. This in no way should disqualify the paper from publication because it is beyond the scope of the experiment (i.e., to establish topographic relationships between perception and memory scene regions in SPL) to demonstrate specific patterns of selectivity/functional relevance of the regions. However, I am curious how the SPL-mem region responds to visually presented scenes. I could imagine that maybe it 1) responds less to visually presented scenes and more to imagined scenes compared to the more posterior perception region or 2) it resembles a task-negative response pattern, such that actively perceiving scenes evokes negative responses while actively remembering evokes positive responses. The first scenario could be interpreted as a graded shift from perception to memory, but still some perceptual processing is happening in the anterior SPL-mem region, while the second scenario would show categorically different types of processing happening in the perception and memory regions. Again, I don't think this type of analysis is necessary for publication, but I invite the authors to respond directly to me and to consider adding something to the manuscript as they see fit.

---

# Reviewer 2

---Summary---

This paper extends a well-established organizational motif in the scene-processing literature to a fourth cortical surface. Prior work from this group and others has shown that the three classical scene-selective regions (OPA, PPA, MPA) are each anatomically paired with an (anterior) memory-responsive counterpart (LPMA, VPMA, MPMA). Recently, a fourth scene-perception region in superior parietal cortex called PIGS was described across several papers. The authors asked whether this fourth perceptual area also has a place-memory counterpart. Using two independent fMRI datasets, they showed that (1) a memory-selective region (SPMA/PIGS-mem) sits immediately anterior and dorsal to SPPA/PIGS in most participants, (2) resting-state functional connectivity dissociates the perception and memory areas into the expected networks, and (3) SPPA/PIGS sits at the confluence of four connectivity branches linking it to regions implicated in egocentric perception and navigation. The authors concluded that the posterior-anterior perception-memory motif is a general organizing principle of scene cortex.

This paper makes a clear and timely contribution to the literature on the cortical organization of scene processing. The recent identification of a fourth scene-selective region in superior parietal cortex (SPPA/PIGS) raised an immediate question of whether this region follows the same posterior-anterior perception-memory organization as the previously described lateral, ventral, and medial surfaces, and the present work addresses that question directly. The demonstration of a memory-responsive region (SPMA/PIGS-mem) anterior and dorsal to SPPA/PIGS provides important clarity about how this newly described fourth perceptual area fits into the broader architecture of scene cortex, and the resting-state connectivity analyses add a complementary line of evidence that the perception and memory areas in superior parietal cortex participate in dissociable functional networks. The paper is also commendable for its use of two independent datasets, with one using static stimuli with a place-versus-people imagery task, and the other using dynamic stimuli with a more demanding place-versus-multiple-social-conditions imagery task. The convergence of findings across these methodologically distinct experiments substantially strengthens confidence in the central result and reduces concerns that the perception-memory pairing reflects features specific to any one task design. The individual-subject localization approach across both datasets is also a clear methodological strength.

That said, I have a few substantive concerns about how the central claim is framed, particularly regarding whether the data fully support the "ubiquitous" four-surface motif emphasized in the abstract and discussion. These concerns do not undermine the new contribution regarding SPPA/PIGS, which I find convincing on its own terms, but I hope the authors will find the following suggestions constructive.

---Major suggestions---

#1

The paper's central conceptual claim is that the posterior-anterior perception-memory motif extends to a fourth cortical surface, supporting a "ubiquitous" coupling between scene-perception and memory-responsive regions. The new evidence for SPPA/PIGS and SPMA/PIGS-mem is convincing on its own terms, and I find the superior parietal results to be the paper's strongest contribution. However, I would encourage the authors to consider whether one specific region is fully supported by the data presented here (and together in Steel 2021, 2025, etc.), particularly with respect to the medial surface (MPA/MPMA). That is, whether MPA and MPMA should be considered functionally separable regions, or an integrated unit representing both scene vision and place memory. I am raising this concern since I consider the perception-memory ROI distinction particularly important for guiding future research conceptualization in vision, memory, and navigation.

By the authors' own reporting, the medial surface behaves differently from the other three. Specifically: (1) in Experiment 1, there was no significant anterior shift of memory relative to perception on the medial surface (F(1,36)=1.26, p=0.27); (2) the Jaccard overlap between MPA and MPMA is significantly higher than on the lateral, ventral, or superior parietal surfaces (Figure 2C); (3) in Experiment 2, the shift was in the opposite direction, with memory falling slightly posterior to perception (p=0.01); and (4) the resting-state connectivity results in Figure 4 also fail to show a clear functional distinction between MPA and MPMA. In the partial correlation matrix (Figure 4A) and the MDS visualization (Figure 4B), MPA and MPMA cluster closely together, and the pattern of their connections to other perception and memory areas appears less differentiated than the analogous pairs on the lateral, ventral, and superior parietal surfaces. Across two independent datasets, then, the medial surface is the one place where the central organizational claim of the paper does not robustly hold. The authors note this and frame it as strengthening the lateral and ventral evidence, but it seems worth engaging with more directly: on the medial surface, the perception-memory pairing motif is either weak or absent in these data.

This has implications for the broader framing. The closing of the discussion argues that "every scene perception area is anatomically coupled to a memory-responsive partner" and that the lateral, ventral, medial, and superior parietal surfaces "all exhibit this posterior-anterior perception-memory motif." Based on the present data, a more accurate summary might be that three of the four surfaces show clear evidence of a discrete perception-memory pairing, while the medial surface shows substantial spatial integration of perception and memory responses without a robust anterior shift. This is not a small distinction - it raises the possibility that the medial surface implements perception-memory integration through a different architectural strategy (overlapping or shared circuitry within a single region) rather than through adjacent paired regions.

I think this would be a more defensible and ultimately more interesting framing, for two reasons. First, it would more accurately represent the gradient of evidence across surfaces shown in the paper itself. Second, the field has increasingly treated MPA and MPMA as cleanly separable functional regions. Given that this paper sits in a position to either reinforce or examine that assumption, I think the authors are well-placed to discuss whether the assumption holds up under scrutiny.

Concretely, I would suggest: (1) tempering the "ubiquitous" / "every scene perception area" language in the abstract and discussion to reflect the cross-surface gradient; (2) adding a paragraph in the discussion that engages directly with why the medial surface behaves differently and what this means for the framework; and (3) considering whether the framework is better described as "three paired surfaces plus one integrated surface" rather than four paired surfaces. None of this undermines the new contribution about SPPA/PIGS. Apparently, MPA/MPMA is not the protagonist of this study, but more accurately contextualizing the findings with regards to this region will better guide future conceptualization and experimentation in this area.

#2

Effect size reporting is inconsistent throughout the paper. Some tests have effect sizes reported while others do not (e.g., most t-tests have Cohen's d reported, while F-tests have no effect sizes reported. Partial eta-squared can be reported with F-tests).

#3

I have some concerns about Figure 4B (MDS visualization) that I would ask the authors to address. First, the MDS is interpretive rather than analytical. The two dimensions are post-hoc labeled as "anatomical surface" and "perception vs. memory," but these are precisely the categorical labels used to define the regions in the first place. The fact that connectivity is structured along these dimensions is therefore not a discovery from the MDS; it is a restatement of how the regions were selected, and is already conveyed by the partial correlation matrix in Figure 4A. Second, no stress value or goodness-of-fit measure is reported. With only 8 regions projected into 2 dimensions, the reader cannot assess how faithfully the projection represents the underlying similarity structure.

More substantively, the patterns in Figure 4B does not straightforwardly support the perception-memory dissociation the authors are emphasizing. On close inspection: (1) MPA and MPMA appear very close together, consistent with my broader concern about the medial surface; (2) SPPA and SPMA also appear close together, closer than one would expect if the "perception vs. memory" dimension were doing strong work for the superior parietal pair; and (3) while OPA and LPMA are more clearly separated along Dimension 2, PPA and VPMA also appear fairly close. In other words, the only pair showing robust separation along the proposed perception-memory dimension is the lateral pair, while the medial, ventral, and superior parietal pairs cluster more tightly. This is the opposite of the impression the figure is presented as conveying, and it again raises questions about the strength of the perception-memory dissociation across surfaces.

I would suggest one of two paths forward. The authors could either (a) report a stress value, clarify whether the dimensional interpretation is data-driven or post-hoc, and engage directly with the pattern of within-pair distances visible in the plot; or (b) remove this panel, since the partial correlation matrix already conveys the same information more quantitatively, and the current MDS visualization risks suggesting a cleaner dissociation than the data actually show.

---Minor suggestions---

#1

The rationale for the four imagery conditions in the Experiment 2 memory task (house, photograph, dancing, conversation) could be more explicitly stated to ease the understanding from naïve readers. As described, the contrast of interest is "place versus all other category simulation trials", which collapses three quite different conditions into a single comparison baseline. These conditions vary along multiple dimensions: imagery modality (visual for photograph and dancing; auditory for conversation), motion content (static for photograph; dynamic for dancing and conversation), and social/self-other content. It is not immediately clear what each non-place condition is intended to control for, or whether the contrast is best interpreted as "scene versus social," "spatial versus non-spatial," or "immersive versus non-immersive imagery." A brief statement in the methods explaining the design logic such as what each condition was meant to dissociate, and why this particular set was chosen, would help readers interpret the memory localizer results and compare them with the simpler place-versus-people contrast used in Experiment 1.

#2

The number of imagery trials per condition in Experiment 2 is not crystal clear upon the first read. The methods describe trial structure (10s simulation, 1s cue, 1s mask, 8s imagery, 4-8s ITI) but I do not see the total number of trials per condition or total scan duration for the memory task.

#3

The "SPPA/PIGS" and "SPMA/PIGS-mem" compound naming is slightly awkward and sometimes inconsistent. Sometimes the paper uses "SPPA/PIGS," sometimes just "PIGS," and "SPMA/PIGS-mem" is a little clunky. The suffix "-mem" appended to "PIGS" effectively means "memory area of the perception area." The authors should either commit to a single name throughout (their own SPMA, the original PIGS, or Yoon et al.'s SPL) and justify it briefly, or at minimum use the compound consistently. Inconsistent abbreviation might cause future researchers to struggle which term(s) to refer to.

Relatedly, in Figure 4 panels A and B, the labels use "SPPA" and "SPMA" without the "/PIGS" suffix, while the text consistently uses "SPPA/PIGS" and "SPMA/PIGS-mem." Worth aligning.

#4

Right above Figure 2 - "Lateral vs Medial: t(10) = -4.478, p = 0.001, d =-1.350, d = 0.20." There are two effect sizes reported here; please fix the typo.

#5

On the medial surface, we observed no anterior shift on the medial surface in Experiment 1, and a tendency for a posterior shift in Experiment 2 (Experiment 1: F(1,36)=1.26, p=0.27; t(9)=0.75, p=0.47, d=0.23; Experiment 2: F(1,42)=6.53, p=0.01, t(10)=-1.08, d=0.33). There seems to be a p-value missing on Experiment 2. Please fix.

#6

"One study elegant study employing a navigation task in identical but visually-distinct environments ..." Please fix the typo by deleting the first "study".

## Author Response

Synthesis of Reviews:

Computational Neuroscience Model Code Accessibility Comments for Author (Required): na Synthesis Statement for Author (Required): # Review Synthesis Both reviewers evaluated this manuscript positively and agree that it makes a clear and timely contribution to the understanding of the cortical organization of scene processing. The analyses are considered thorough, and the manuscript is well written. The suggestions from both reviewers are relatively minor in nature, consisting mainly of requests for clarification and elaboration. Reviewer #2 raises one more substantive point regarding the framing of the medial surface results and the "ubiquitous" motif claim, which should be addressed in the discussion.

In the post-review consultation, both reviewers agreed that the MDS analysis (Figure 4B) is difficult to interpret as presented and should be clarified or reconsidered. Several of the points raised by Reviewer #1 may be addressable in the reply letter alone; the authors may decide whether they prefer to revise the manuscript accordingly.

We thank the editors and the reviewers for the constructive feedback on our manuscript. The reviewers provided excellent suggestions, and we believe that our work has been much improved through revision. In particular, we made the following changes: ● Simplified our ROI naming convention. We now refer to the areas as SPA (Superior place area) and SPMA (Superior place memory area), which better aligns with the larger scene perception literature. ● Added a discussion of the lack of topographic shift between MPA/MPMA (R2, C1).

This included tempering our overall claim of "ubiquitous shifting" in the abstract and discussion. ● Added an analysis of the memory areas' activity during perception (R1, C3). This revealed significant selectivity during perception of dynamic visual scenes. This complements the observation of anterior shifted memory activity. ● Added an analysis of connectivity from SPMA (R1, C2). This revealed an intriguing parallel between the streams of connectivity from SPA and SPMA. ● Report effect sizes and confirmed all statistical analyses.

In the process of confirming our statistical analyses, we identified an error in the connectivity code, which referenced an outdated ROI set. This affected only the connectivity analysis.

Correcting it left all effects qualitatively unchanged, with no changes in significance and slightly larger effect sizes throughout. # Reviewer 1 The authors ran two separate experiments to demonstrate that a visual scene-selective region in the superior parietal lobe (SPL) follows the same perception-memory motif as the other cortical scene regions (i.e., OPA, PPA, MPA). Specifically, the authors showed participants static (i.e., pictures) scenes and faces (experiment 1), and dynamic (i.e., short videos) scenes, faces, bodies, and objects (experiment 2). They defined the perceptual scene-selective region in SPL (and the other cortical scene regions) using the contrast, scenes > than non-scene stimuli. In addition to these visual stimuli, participants were also asked to imagine the different categories and the authors again used the same contrast to look for the memory-specific scene region in SPL (and the other cortical scene regions). In both datasets the authors found that predicted perception memory motif in SPL - i.e., recalling scenes evoked responses immediately anterior and dorsal to the region responding to the visually presented scene stimuli. In addition to the localization of the memory-responsive SPL scene region (SPL-mem), the authors, include an analysis of how it relates to retinotopic maps in the parietal lobe and a resting-state analysis to show how the SPL scene region is connected to the rest of the cortical scene network.

The analyses are reasonable and thorough, and the manuscript is clearly written.

In fact, I find very little to critique in this manuscript. Below are some minor suggestions that the authors might choose to address in the final draft.

We thank the reviewer for their thoughtful comments. We have incorporated their suggestions throughout the manuscript.

1. SPL localization in each hemisphere. The criteria you used for SPL definition was not clear to me. You mention that you found SPL bilaterally in almost every participant. Did you exclude participants if you found SPL in only one hemisphere or did you include them since you found no differences between hemispheres anyway? You mention that the other "regions of interest were present in at least one hemisphere". Did you use different criteria for the SPL and the other ROIs? A little clarification in the revised manuscript could be helpful.

The reviewer asks for clarification on the definition of SPA and SPMA.

All ROIs, including SPA were defined using the contrast of scenes versus faces. For SPA, we incorporated the anatomical definition described in Kennedy et al., 2024 and Yoon et al., 2025. All participants in whom we could define SPA in at least one hemisphere were included in the analyses; we used linear mixed effects modeling versus a repeated measures ANOVA to better account for participants in whom we failed to define regions bilaterally.

We have added the following text to clarify this point.

Scene perception areas (OPA, PPA, MPA, and SPA) were defined for each participant by contrasting activity from scene versus face blocks (t-stat > 2.79 (p<0.005), surface cluster size > 20). (Pg.

9, Line 307) Scene perception areas (OPA, PPA, MPA, and SPA) were defined for each participant by contrasting activity from scene versus face blocks (t-stat > 2.79 (p<0.005), surface cluster size (k) > 20). (Pg.

9, Line 343) We used linear mixed effects modeling to account for participants in whom we could only identify regions in one hemisphere more effectively. (Pg.

10, Line 375) 2. Connectivity from the SPL-mem? The resting-state functional connectivity analysis uses the scene perception region in SPL as a seed. Why not do the same for the SPL-mem? The reviewer raises an interesting point regarding the connectivity of SPMA. We did not explore this in our original manuscript because of the anatomical adjacency of SPA and SPMA at the posterior confluence of the superior occipital cortex and the precuneus.

The reviewer's astute comment prompted us to take a closer look at the distinction between these regions' functional connectivity. This revealed additional nuance to our original finding: the connectivity pathway from SPA through the IPS retinotopic maps is paralleled along its medial and lateral portions by two distinct pathways of increased connectivity from SPMA. We include further detail of this finding below, and in the revised version of Figure 4 (now Figure 5).

For this analysis, we performed the same method presented for SPA on SPMA, in which we calculated the 90% most connected vertices to SPMA for each participant. We then averaged these individual preference maps across participants to form probability maps, and we qualitatively compared the resulting maps.

Response figure 1. Parallel branching connectivity streams within the superior parietal cortex emanating from SPA (left) and SPMA (middle). In each participant, we calculated whole-brain connectivity of SPA and SPMA, then created binarized masks of the 90th percentile of connected vertices. We aggregated these masks across participants to create a probabilistic map of SPA and SPMA connectivity. This analysis revealed parallel streams of connectivity spanning superior parietal cortex and intraparietal sulcus. Connectivity from SPA largely follows IPS, with branches emanating 1) posterior medially (toward MPA/MPMA), 2) posterior ventrally (toward OPA/LPMA), 3) dorsal medially (toward marginal ramus of the cingulate, places-2), and 4) dorsal laterally (toward parietal area PF). Connectivity from SPMA borders these connectivity streams on the lateral and medial sides, with peaks of connectivity falling anterior to the peaks of SPA connectivity at each branch's terminus. The right panel depicts the SPA and SPMA probability maps, showing vertices that exceeded a 60% overlap across participants.

We think this result is complementary to our previous finding, and adds important elaboration. Clearly, connectivity from the perception and memory areas form integrated branches within medial and lateral parietal cortex; this is present in both SPA and SPMA connectivity maps. The peaks of connectivity correspond to multiple place memory/scene perception areas, consistent with the greater connectivity from among the memory and perception areas revealed in the network analysis. Importantly, these connectivity branches run in parallel, suggesting paired and related systems. Finally, these streams come together within IPS, suggesting this area is an anatomical confluence for perceptual and memory-related processes.

We have added this figure to the manuscript and have revised the manuscript to discuss the significance of this finding.

This analysis revealed that connectivity from SPA and SPMA form parallel connectivity pathways extending throughout parietal cortex. SPA sits at the posterior confluence of four anatomically distinct branches of preferential connectivity extending into the dorsal parietal cortex. These branches are bordered on their medial and lateral sides by areas with high connectivity with SPMA. Figure 5C shows the proportion of overlapping participants connectivity maps from SPA (Left), SPMA (middle), and the overlap between these maps thresholded at 60% overlapping participants (right).

Ventrally, connectivity from SPA follows two distinct branches. Branch 1 runs laterally, connecting SPA to OPA. Branch 2 runs medially, connecting SPA to MPA/MPMA via the parieto-occipital sulcus. These connectivity streams converge at SPA and form a single stream moving anterior and dorsally, through SPMA toward superior parietal cortex. Both branches are bordered by increased connectivity with SPMA, the lateral stream beginning in LPMA, and the medial stream beginning in MPA/MPMA.

Dorsally, the connectivity stream emanating from SPA splits into two additional dorsal medial and lateral branches. In the group average map, the bifurcation appeared within the superior aspect of the marginal ramus of the cingulate and the retinotopic map SPL1. At the bifurcation point, Branch 3 dove down the medial wall toward the inflection point of the marginal ramus of the cingulate. Branch 4 extended ventrally and laterally into PF complex and area PFt in lateral parietal cortex(Glasser et al., 2016). The end points of branches 1-3 have recognized roles in navigation-related processes (Marchette et al., 2014; Persichetti and Dilks, 2018; Zhang et al., 2025), and area PF/PFt has recently been identified as a region of a larger brain network related to navigation(Zhang et al., 2025). Connectivity from SPMA followed these branches toward their termini, paralleling their medial and lateral trajectories. This positions the superior parietal cortex as an anatomical hub connecting disparate cortical areas to facilitate visually-guided navigation.

In addition to these parietal connectivity pathways in superior parietal cortex, we observed three other loci of increased SPA and SPMA connectivity distributed in cortex. Interestingly, these areas tended to be at the anterior edges of retinotopic maps(Wang et al., 2015), and at each location, SPMA connectivity fell at the anterior edge of the SPA connectivity cluster. One patch of connectivity in medial ventral temporal cortex, encompassing the retinotopic maps TE1&2 and PHC1&2, consistent with the location of PPA and VPMA. A second patch of connectivity was in lateral occipitotemporal cortex, anterior to the retinotopic maps MT/hMT. Finally, we found a third patch in lateral prefrontal cortex, anterior to the frontal eye fields. These connectivity peaks at anterior portions of the visual retinotopic maps suggest a broader linkage between areas at the boundary of classically-defined visual areas. (Page 17-18, Lines 647-680) SPA sits at a junction of four distinct connectivity branches: two medial branches between the parieto-occiptal cortex and dorsal precuneus, and two lateral branches between lateral occipital and parietal cortices. These branches were bordered on by two parallel streams of connectivity to SPMA, with each the SPMA stream's endpoint falling anterior to SPA's. This finding reinforces two key points. This finding reinforces two key points. First, these results show that perception and memory processes are intimately paired throughout cortex, and superior parietal cortex appears to be an area in which these processes come together. Second, they further support the expanded role of superior parietal cortex in navigation function. Importantly, these branches span regions implicated in navigation-relevant processes, including processes related to allocentric map-like navigation(Marchette et al., 2014; Persichetti and Dilks, 2018; Lu et al., 2025; Naveilhan et al., 2025), perspective taking(Zhang et al., 2025), and ego-centric scene perception(Pitcher et al., 2011; Persichetti and Dilks, 2016, 2019). Being situated at the intersection of these streams puts superior parietal cortex, home to SPA and SPMA, in a unique position as an integrative hub, where our current percept is connected with our internal representation of location and current goal to guide our attention and inform our movement. (Page 20, Lines 769-783).

Figure 5. Functional connectivity during rest dissociates perception and memory areas in superior parietal cortex. A. Pairwise partial correlation(Smith et al., 2013) between scene perception areas (OPA, PPA, MPA, SPPA) and place memory areas (LMPA, VPMA, MPMA, SPMA), providing an estimate of the unique variance explained at each connection. B.

Differential connectivity between SPPA/PIGS and SPMA/PIGS-mem with the other scene perception and place memory areas. SPPA/PIGS was more connected with other scene perception areas, while SPMA/PIGS-mem is more connected with the place memory areas. C.

Parallel branching connectivity streams within the superior parietal cortex emanating from SPA (left) and SPMA (middle). In each participant, we calculated whole-brain connectivity of SPA and SPMA, then created binarized masks of the 90th percentile of connected vertices. We aggregated these masks across participants to create a probabilistic map of SPA and SPMA connectivity. This analysis revealed parallel streams of connectivity spanning superior parietal cortex and intraparietal suclus. Connectivity from SPA largely follows IPS, with branches emanating 1) posterior medially (toward MPA/MPMA), 2) posterior ventrally (toward OPA/LPMA), 3) dorsal medially (toward marginal ramus of the cingulate, places-2), and 4) dorsal laterally (toward parietal area PF). Connectivity from SPMA borders these connectivity streams on the lateral and medial sides, with peaks of connectivity falling anterior to the peaks of SPA connectivity at each branch's terminus. The right panel shows the overlap between the two probability maps, thresholded at 60% overlapping participants at each vertex.

3. How does SPL-mem respond to visually presented scenes? The only shortcoming of this paper is that it does not include an independent set of conditions to probe the selectivity of the functionally defined ROIs. This in no way should disqualify the paper from publication because it is beyond the scope of the experiment (i.e., to establish topographic relationships between perception and memory scene regions in SPL) to demonstrate specific patterns of selectivity/functional relevance of the regions. However, I am curious how the SPL-mem region responds to visually presented scenes. I could imagine that maybe it 1) responds less to visually presented scenes and more to imagined scenes compared to the more posterior perception region or 2) it resembles a task-negative response pattern, such that actively perceiving scenes evokes negative responses while actively remembering evokes positive responses. The first scenario could be interpreted as a graded shift from perception to memory, but still some perceptual processing is happening in the anterior SPL-mem region, while the second scenario would show categorically different types of processing happening in the perception and memory regions. Again, I don't think this type of analysis is necessary for publication, but I invite the authors to respond directly to me and to consider adding something to the manuscript as they see fit.

The reviewer raises an interesting point regarding how the memory areas respond to visually presented scenes. Overall, we found evidence that the place memory areas are active during scene perception in a relatively selective manner. This suggests that although these regions are preferentially active during memory tasks, they retain their selectivity when perceiving dynamic visual scenes.

For this analysis, we extracted the activity estimates (beta-values) from the place memory areas (including SPL) in the dynamic localizer task collected during Experiment 2 (Dartmouth). We focused on the Dartmouth dataset because this experiment included rest blocks (12s), allowing us to assess the activation during perception versus a fixation baseline. For this analysis, we considered the 300 nodes around the center of mass.

Across all ROIs, we observed a Main effect of Category (LPMA: F(3,76)=12.96, p<0.001, eta2=0.334; VPMA: F(3,76)=33.973, p<0.001, eta2=0.573; MPMA:

F(3,76)=20.708, p<0.001, eta2=0.437; SPMA: F(3,68)=8.931, p<0.001, eta2=0.283).

Critically, scenes evoked greater activity compared with all other categories in LPMA, VPMA, and MPMA (ts>3.41, p-valuescorrected<0.04). The notable exception was SPMA, where we observed a significant difference between activity during scene perception compared with faces (t(9)=5.395, pcorrected=0.003, d=1.70) and bodies (t(9)=3.986, pcorrected=0.019, d=1.26), but not objects (t(9)=1.934, pcorrected=0.511, d=0.612).

As the reviewer points out, our manuscript primarily centers on the shift in activity. We think this result adds important nuance to our results but does not alter our overall interpretation.

We have included this result as a figure in the revised manuscript.

Figure 4. The place memory areas activate in a relatively selective manner to visually-presented scenes. We extracted activation (beta-values) during the dynamic perception task from the place memory areas in participants from Study 2 (Dartmouth). All place memory areas showed greater activity during scene perception compared with other categories (faces and bodies), although SPMA was not significantly different from objects. This is consistent with the general preference for spatial information, even in the absence of memory recall demands.

Our prior analysis shows that recalling personally-familiar places activates brain areas anterior to the scene perception areas. A related question is whether the place memory areas retain their selectivity when perceiving visual scenes. To address this question, we extracted the activity estimates (beta-values) from the place memory areas (including SPA) in the dynamic localizer task collected during Experiment 2 (Dartmouth). We focused on the Dartmouth dataset because this experiment included rest blocks (12s), allowing us to assess the activation during perception versus a fixation baseline. To equate SNR and signal homogeneity across the regions, we constrained these regions to the 300 vertices closest to each region's center of mass.

We found that the place memory areas activated in a relatively selective manner during scene perception, consistent with the partial overlap between the scene perception and place memory areas (Figure 4). Across all ROIs, we observed a Main effect of Category (LPMA:

F(3,76)=12.96, p<0.001, eta2=0.334; VPMA: F(3,76)=33.973, p<0.001, eta2=0.573; MPMA:

F(3,76)=20.708, p<0.001, eta2=0.437; SPMA: F(3,68)=8.931, p<0.001, eta2=0.283). Critically, viewing scenes evoked greater activity compared with all other categories in LPMA, VPMA, and MPMA (ts>3.41, ps<0.04). The notable exception was SPMA, where we observed a significant difference between activity during scene perception compared with faces (t(9)=5.395, p=0.003, d=1.70) and bodies (t(9)=3.986, p=0.019, d=1.26), but not objects (t(9)=1.934, p=0.511, d=0.612). This suggests that although these regions are preferentially active during memory tasks, they retain their selectivity when perceiving dynamic visual scenes. (Pg. 16, Lines 569-588) Using mental imagery of scenes to isolate top-down processes, we found that SPA falls at the posterior boundary of a larger memory-responsive cluster extending through precuneus toward the marginal ramus of the cingulate. We could localize this memory-responsive region, SPMA, in the majority of participants across two studies despite different tasks and experimental parameters, which speaks to its robustness. Like the other memory responsive regions (Steel et al., 2021, 2025), SPMA was anatomically distinct from its perceptual counterpart, with limited overlap between these functionally-defined areas. Interestingly, when viewing dynamic stimuli, SPMA retained its scene selectivity, suggesting that the preference for scenes in memory extends into perception in these areas at a sub-threshold level. Although this pattern may be driven by the small overlapping area between these functiona regions of interest, further work should continue to resolve the functional dissociation of the perception and memory areas. The ability to localize SPA and SPMA in individual participants suggests that they are meaningful features of brain organization(Petersen et al., 2024) and opens up the possibility of studying these functional areas' specific roles in coordinating scene perception and visuospatial memory. (Pg. 19, Lines 716-729) --- # Reviewer 2 ---Summary--- This paper extends a well-established organizational motif in the scene-processing literature to a fourth cortical surface. Prior work from this group and others has shown that the three classical scene-selective regions (OPA, PPA, MPA) are each anatomically paired with an (anterior) memory-responsive counterpart (LPMA, VPMA, MPMA). Recently, a fourth scene-perception region in superior parietal cortex called PIGS was described across several papers. The authors asked whether this fourth perceptual area also has a place-memory counterpart. Using two independent fMRI datasets, they showed that (1) a memory-selective region (SPMA/PIGS-mem) sits immediately anterior and dorsal to SPPA/PIGS in most participants, (2) resting-state functional connectivity dissociates the perception and memory areas into the expected networks, and (3) SPPA/PIGS sits at the confluence of four connectivity branches linking it to regions implicated in egocentric perception and navigation. The authors concluded that the posterior-anterior perception-memory motif is a general organizing principle of scene cortex.

This paper makes a clear and timely contribution to the literature on the cortical organization of scene processing. The recent identification of a fourth scene-selective region in superior parietal cortex (SPPA/PIGS) raised an immediate question of whether this region follows the same posterior-anterior perception-memory organization as the previously described lateral, ventral, and medial surfaces, and the present work addresses that question directly. The demonstration of a memory-responsive region (SPMA/PIGS-mem) anterior and dorsal to SPPA/PIGS provides important clarity about how this newly described fourth perceptual area fits into the broader architecture of scene cortex, and the resting-state connectivity analyses add a complementary line of evidence that the perception and memory areas in superior parietal cortex participate in dissociable functional networks. The paper is also commendable for its use of two independent datasets, with one using static stimuli with a place-versus-people imagery task, and the other using dynamic stimuli with a more demanding place-versus-multiple-social-conditions imagery task. The convergence of findings across these methodologically distinct experiments substantially strengthens confidence in the central result and reduces concerns that the perception-memory pairing reflects features specific to any one task design. The individual-subject localization approach across both datasets is also a clear methodological strength.

That said, I have a few substantive concerns about how the central claim is framed, particularly regarding whether the data fully support the "ubiquitous" four-surface motif emphasized in the abstract and discussion. These concerns do not undermine the new contribution regarding SPPA/PIGS, which I find convincing on its own terms, but I hope the authors will find the following suggestions constructive.

We appreciate the reviewer's careful consideration of our work. We have revised the manuscript and paid careful consideration to the scope of our claims. We feel that the manuscript is much improved from these suggestions. ---Major suggestions--- #1 The paper's central conceptual claim is that the posterior-anterior perception-memory motif extends to a fourth cortical surface, supporting a "ubiquitous" coupling between scene-perception and memory-responsive regions. The new evidence for SPPA/PIGS and SPMA/PIGS-mem is convincing on its own terms, and I find the superior parietal results to be the paper's strongest contribution. However, I would encourage the authors to consider whether one specific region is fully supported by the data presented here (and together in Steel 2021, 2025, etc.), particularly with respect to the medial surface (MPA/MPMA). That is, whether MPA and MPMA should be considered functionally separable regions, or an integrated unit representing both scene vision and place memory. I am raising this concern since I consider the perception-memory ROI distinction particularly important for guiding future research conceptualization in vision, memory, and navigation.

By the authors' own reporting, the medial surface behaves differently from the other three. Specifically: (1) in Experiment 1, there was no significant anterior shift of memory relative to perception on the medial surface (F(1,36)=1.26, p=0.27); (2) the Jaccard overlap between MPA and MPMA is significantly higher than on the lateral, ventral, or superior parietal surfaces (Figure 2C); (3) in Experiment 2, the shift was in the opposite direction, with memory falling slightly posterior to perception (p=0.01); and (4) the resting-state connectivity results in Figure 4 also fail to show a clear functional distinction between MPA and MPMA.

In the partial correlation matrix (Figure 4A) and the MDS visualization (Figure 4B), MPA and MPMA cluster closely together, and the pattern of their connections to other perception and memory areas appears less differentiated than the analogous pairs on the lateral, ventral, and superior parietal surfaces. Across two independent datasets, then, the medial surface is the one place where the central organizational claim of the paper does not robustly hold. The authors note this and frame it as strengthening the lateral and ventral evidence, but it seems worth engaging with more directly: on the medial surface, the perception-memory pairing motif is either weak or absent in these data.

This has implications for the broader framing. The closing of the discussion argues that "every scene perception area is anatomically coupled to a memory-responsive partner" and that the lateral, ventral, medial, and superior parietal surfaces "all exhibit this posterior-anterior perception-memory motif." Based on the present data, a more accurate summary might be that three of the four surfaces show clear evidence of a discrete perception-memory pairing, while the medial surface shows substantial spatial integration of perception and memory responses without a robust anterior shift. This is not a small distinction - it raises the possibility that the medial surface implements perception-memory integration through a different architectural strategy (overlapping or shared circuitry within a single region) rather than through adjacent paired regions.

I think this would be a more defensible and ultimately more interesting framing, for two reasons. First, it would more accurately represent the gradient of evidence across surfaces shown in the paper itself. Second, the field has increasingly treated MPA and MPMA as cleanly separable functional regions.

Given that this paper sits in a position to either reinforce or examine that assumption, I think the authors are well-placed to discuss whether the assumption holds up under scrutiny.

Concretely, I would suggest: (1) tempering the "ubiquitous" / "every scene perception area" language in the abstract and discussion to reflect the cross-surface gradient; (2) adding a paragraph in the discussion that engages directly with why the medial surface behaves differently and what this means for the framework; and (3) considering whether the framework is better described as "three paired surfaces plus one integrated surface" rather than four paired surfaces. None of this undermines the new contribution about SPPA/PIGS.

Apparently, MPA/MPMA is not the protagonist of this study, but more accurately contextualizing the findings with regards to this region will better guide future conceptualization and experimentation in this area.

We thank the reviewer for their deep consideration of our framework and their insightful discussion of how MPA fits with the idea of a distinction between perception and memory processes. We agree with the reviewer that the original framing overstates the consistency of the perception-memory pairing across the four cortical surfaces.

In response to the reviewer, we have made the following changes. First, we have removed the word "ubiquitous" and related terms/phrases from the abstract and discussion, and softened this point overall. We feel this softening brings the claims more in line with our findings here and in previous papers.

Second, we have added a new discussion paragraph that addresses the medial surface's distinct pattern compared with the lateral, ventral, and superior surfaces. We frame this as an empirical question and present two possibilities that we think are consistent with the present literature: 1) MPA/MPMA constitute a unified perception-memory area as the reviewer suggests, and 2) a distinction exists but is not captured by the anterior-posterior heuristic we have employed here. We think that adjudicating between these possibilities will be an interesting target for future investigations.

We have made the following revisions to the manuscript.

Abstract Visual scene analysis relies on a set of scene-selective regions in posterior cerebral cortex (OPA, PPA, MPA), with a paired memory-responsive counterpart located in anterior (LPMA, VPMA) or overlapping cortex (MPMA). (Pg.

2, Lines 36-37) The widespread anatomical coupling between scene-perception and memory processes reflects the importance of this interaction for flexible, context-grounded navigation. (Pg. 2, Lines 49-51) Discussion In the context of scenes, prior work has shown that co-localization of perceptual and memory-related brain areas facilitates their interaction: immediately anterior to the lateral and ventral scene perception areas exists a memory-responsive region that represents visuospatial context currently out of view(Silson et al., 2016b, 2019a; Steel et al., 2021, 2023, 2025; Srokova et al., 2022). (Pg. 18, Lines 682-698) The distinction between perception and memory is largely consistent on the lateral, ventral, and superior parietal surfaces. The medial surface responds strongly to mnemonic and spatial context, including familiarity with scenes and spatial knowledge (Bar and Aminoff, 2003; Sugiura et al., 2005; Epstein et al., 2007a, 2007b; Park and Chun, 2009; Robertson et al., 2016;

Persichetti and Dilks, 2018, 2019; Berens et al., 2021). One possibility is that this surface constitutes a unified perception-memory architecture rather than a topographically distinct pairing. We did not observe a consistent anterior shift from perception to memory on the medial surface, consistent with other work that has also failed to observe this effect (Steel et al., 2025), and our prior work found similar responses to increasing spatial knowledge during perception and imagery in MPA and MPMA (Steel et al., 2023). A second possibility is a more complex distinction not well captured by the simple anterior-shift heuristic. For example, other work has observed a perception-memory distinction on the medial surface, including distinct peaks in activity across the boundary of the parieto-occipital cortex (Silson et al., 2019a) and intermixed positive and negative retinotopic coding in this area (Scrivener and Silson, 2025). Resolving this will likely require targeted investigation of medial parietal cortex, including tasks designed specifically to engage this region and high-resolution fMRI to disambiguate the anterior and posterior banks of the parieto-occipital sulcus. (Pg. 19, Lines 730-746) #2 Effect size reporting is inconsistent throughout the paper. Some tests have effect sizes reported while others do not (e.g., most t-tests have Cohen's d reported, while F-tests have no effect sizes reported. Partial eta-squared can be reported with F-tests).

We have now added partial eta sq associated with all ANOVAs throughout the results. #3 I have some concerns about Figure 4B (MDS visualization) that I would ask the authors to address. First, the MDS is interpretive rather than analytical. The two dimensions are post-hoc labeled as "anatomical surface" and "perception vs. memory," but these are precisely the categorical labels used to define the regions in the first place. The fact that connectivity is structured along these dimensions is therefore not a discovery from the MDS; it is a restatement of how the regions were selected, and is already conveyed by the partial correlation matrix in Figure 4A. Second, no stress value or goodness-of-fit measure is reported. With only 8 regions projected into 2 dimensions, the reader cannot assess how faithfully the projection represents the underlying similarity structure.

More substantively, the patterns in Figure 4B does not straightforwardly support the perception-memory dissociation the authors are emphasizing. On close inspection: (1) MPA and MPMA appear very close together, consistent with my broader concern about the medial surface; (2) SPPA and SPMA also appear close together, closer than one would expect if the "perception vs. memory" dimension were doing strong work for the superior parietal pair; and (3) while OPA and LPMA are more clearly separated along Dimension 2, PPA and VPMA also appear fairly close. In other words, the only pair showing robust separation along the proposed perception-memory dimension is the lateral pair, while the medial, ventral, and superior parietal pairs cluster more tightly. This is the opposite of the impression the figure is presented as conveying, and it again raises questions about the strength of the perception-memory dissociation across surfaces.

I would suggest one of two paths forward. The authors could either (a) report a stress value, clarify whether the dimensional interpretation is data-driven or post-hoc, and engage directly with the pattern of within-pair distances visible in the plot; or (b) remove this panel, since the partial correlation matrix already conveys the same information more quantitatively, and the current MDS visualization risks suggesting a cleaner dissociation than the data actually show.

We agree with the reviewer's assessment, and we have removed this panel from the manuscript. ---Minor suggestions--- #1 The rationale for the four imagery conditions in the Experiment 2 memory task (house, photograph, dancing, conversation) could be more explicitly stated to ease the understanding from naïve readers. As described, the contrast of interest is "place versus all other category simulation trials", which collapses three quite different conditions into a single comparison baseline. These conditions vary along multiple dimensions: imagery modality (visual for photograph and dancing; auditory for conversation), motion content (static for photograph; dynamic for dancing and conversation), and social/self-other content. It is not immediately clear what each non-place condition is intended to control for, or whether the contrast is best interpreted as "scene versus social," "spatial versus non-spatial," or "immersive versus non-immersive imagery." A brief statement in the methods explaining the design logic such as what each condition was meant to dissociate, and why this particular set was chosen, would help readers interpret the memory localizer results and compare them with the simpler place-versus-people contrast used in Experiment 1.

We have added the following information to the methods:

Data from Dartmouth were collected as part of a study investigating spatial and social imagery.

Prior to scanning, participants provided the names of six people for whom they could imagine the following four prompts: i) imagine a room in that person's house, ii) a specific photograph of that person, iii) that person dancing, and iv) having a conversation with that person. These people and prompts served as cues for the spatial versus social imagery task. Here, we only considered the contrast of spatial compared with the mixed social conditions; the results specific to the social imagery conditions will be discussed in a separate report. Data from Dartmouth were collected as part of a study investigating spatial and social imagery. Prior to scanning, participants provided the names of six people for whom they could imagine the following four prompts: i) imagine a room in that person's house, ii) a specific photograph of that person, iii) that person dancing, and iv) having a conversation with that person. These people and prompts served as cues for the spatial versus social imagery task. Here, we only considered the contrast of spatial compared with the mixed social conditions; the results specific to the social imagery conditions will be discussed in a separate report. (Pg. 5-6, Lines 185-191) #2 The number of imagery trials per condition in Experiment 2 is not crystal clear upon the first read. The methods describe trial structure (10s simulation, 1s cue, 1s mask, 8s imagery, 4-8s ITI) but I do not see the total number of trials per condition or total scan duration for the memory task.

We appreciate the reviewer pointing out this omission. We have added this information to the methods.

Participants underwent 4 runs of fMRI scanning on the social imagery task. Each stimulus x condition pairing was shown once per run (4 stimuli per condition x 4 conditions = 16 trials per run). Each scanning run lasted 280s (140 TRs). (Pg. 6, Lines 217-219) #3 The "SPPA/PIGS" and "SPMA/PIGS-mem" compound naming is slightly awkward and sometimes inconsistent. Sometimes the paper uses "SPPA/PIGS," sometimes just "PIGS," and "SPMA/PIGS-mem" is a little clunky. The suffix "-mem" appended to "PIGS" effectively means "memory area of the perception area." The authors should either commit to a single name throughout (their own SPMA, the original PIGS, or Yoon et al.'s SPL) and justify it briefly, or at minimum use the compound consistently. Inconsistent abbreviation might cause future researchers to struggle which term(s) to refer to.

Relatedly, in Figure 4 panels A and B, the labels use "SPPA" and "SPMA" without the "/PIGS" suffix, while the text consistently uses "SPPA/PIGS" and "SPMA/PIGS-mem." Worth aligning.

We appreciate the reviewer's suggestion. We have adopted the labels "SPA" and "SPMA" throughout the manuscript. This better matches the convention of the other perception and memory areas, and aligns with recent publications from recent work describing these regions (Jung et al., 2026, bioRxiv). #4 Right above Figure 2 - "Lateral vs Medial: t(10) = -4.478, p = 0.001, d =-1.350, d = 0.20." There are two effect sizes reported here; please fix the typo.

Fixed. #5 On the medial surface, we observed no anterior shift on the medial surface in Experiment 1, and a tendency for a posterior shift in Experiment 2 (Experiment 1:

F(1,36)=1.26, p=0.27; t(9)=0.75, p=0.47, d=0.23; Experiment 2: F(1,42)=6.53, p=0.01, t(10)=-1.08, d=0.33). There seems to be a p-value missing on Experiment 2. Please fix. #6 "One study elegant study employing a navigation task in identical but visually-distinct environments ..." Please fix the typo by deleting the first "study".

Fixed.

## References

[B1] Aguirre GK, D’Esposito M (1999) Topographical disorientation: a synthesis and taxonomy. Brain 122:1613–1628. 10.1093/brain/122.9.1613 10468502

[B2] Angeli PA, Steel A, Silson EH, Robertson CE (2024) Positive and negative retinotopic codes in the human hippocampus. bioRxiv:2024.09.27.615397.

[B3] Auger SD, Mullally SL, Maguire EA (2012) Retrosplenial cortex codes for permanent landmarks. PLoS One 7:e43620. 10.1371/journal.pone.0043620 22912894 PMC3422332

[B4] Auger SD, Zeidman P, Maguire EA (2015) A central role for the retrosplenial cortex in de novo environmental learning. eLife 4:e09031. 10.7554/eLife.09031 26284602 PMC4559753

[B5] Bar M, Aminoff E (2003) Cortical analysis of visual context. Neuron 38:347–358. 10.1016/S0896-6273(03)00167-3 12718867

[B6] Bartnik CG, Sartzetaki C, Sanchez AP, Molenkamp E, Bommer S, Vukšić N, Groen IIA (2025) Representation of locomotive action affordances in human behavior, brains, and deep neural networks. Proc Natl Acad Sci U S A 122:e2414005122. 10.1073/pnas.2414005122 40504155 PMC12184334

[B7] Berens SC, Joensen BH, Horner AJ (2021) Tracking the emergence of location-based spatial representations in human scene-selective cortex. J Cogn Neurosci 33:445–462. 10.1162/jocn_a_01654 33284080 PMC8658499

[B8] Biswal B, Zerrin Yetkin F, Haughton VM, Hyde JS (1995) Functional connectivity in the motor cortex of resting human brain using echo-planar MRI. Magn Reson Med 34:537–541. 10.1002/mrm.1910340409 8524021

[B9] Bonner MF, Epstein RA (2017) Coding of navigational affordances in the human visual system. Proc Natl Acad Sci U S A 114:4793–4798. 10.1073/pnas.1618228114 28416669 PMC5422815

[B10] Breedlove JL, St-Yves G, Olman CA, Naselaris T (2020) Generative feedback explains distinct brain activity codes for seen and mental images. Curr Biol 30:2211–2224. 10.1016/j.cub.2020.04.014 32359428 PMC10230322

[B11] Cox RW (1996) AFNI: software for analysis and visualization of functional magnetic resonance neuroimages. Comput Biomed Res 29:162–173. 10.1006/cbmr.1996.0014 8812068

[B12] Dickie EW, Ameis SH, Shahab S, Calarco N, Smith DE, Miranda D, Viviano JD, Voineskos AN (2018) Personalized intrinsic network topography mapping and functional connectivity deficits in autism spectrum disorder. Biol Psychiatry 84:278–286. 10.1016/j.biopsych.2018.02.1174 29703592 PMC6076333

[B13] Dilks DD, Julian JB, Paunov AM, Kanwisher N (2013) The occipital place area is causally and selectively involved in scene perception. J Neurosci 33:1331–1336. 10.1523/JNEUROSCI.4081-12.2013 23345209 PMC3711611

[B14] Dilks DD, Kamps FS, Persichetti AS (2022) Three cortical scene systems and their development. Trends Cogn Sci 26:117–127. 10.1016/j.tics.2021.11.002 34857468 PMC8770598

[B15] DuPre E, Salo T, Markello R, Kundu P, Whitaker K, Handwerker D (2019) ME-ICA/tedana: 0.0.6. Available at: https://zenodo.org/record/2558498 [Accessed February 8, 2020].

[B16] DuPre E, et al. (2021) TE-dependent analysis of multi-echo fMRI with *tedana*. J Open Source Softw 6:3669. 10.21105/joss.03669

[B17] Dwivedi K, Cichy RM, Roig G (2020) Unraveling representations in scene-selective brain regions using scene-parsing deep neural networks. J Cogn Neurosci 33:2032–2043. 10.1162/jocn_a_01624 PMC7612022 32897121

[B20] Epstein RA, Baker CI (2019) Scene perception in the human brain. Annu Rev Vis Sci 5:373–397. 10.1146/annurev-vision-091718-014809 31226012 PMC6989029

[B21] Epstein R, Kanwisher N (1998) A cortical representation of the local visual environment. Nature 392:598–601. 10.1038/33402 9560155

[B18] Epstein RA, Higgins JS, Jablonski K, Feiler AM (2007a) Visual scene processing in familiar and unfamiliar environments. J Neurophysiol 97:3670–3683. 10.1152/jn.00003.2007 17376855

[B19] Epstein RA, Parker WE, Feiler AM (2007b) Where am I now? Distinct roles for parahippocampal and retrosplenial cortices in place recognition. J Neurosci 27:6141–6149. 10.1523/JNEUROSCI.0799-07.2007 17553986 PMC6672165

[B22] Favila SE, Lee H, Kuhl BA (2020) Transforming the concept of memory reactivation. Trends Neurosci 43:939–950. 10.1016/j.tins.2020.09.006 33041061 PMC7688497

[B23] Favila SE, Kuhl BA, Winawer J (2022) Perception and memory have distinct spatial tuning properties in human visual cortex. Nat Commun 13:1–21. 10.1038/s41467-022-33161-8 36257949 PMC9579130

[B24] Fischl B (2012) FreeSurfer. Neuroimage 62:774–781. 10.1016/j.neuroimage.2012.01.021 22248573 PMC3685476

[B25] Friston KJ, Holmes AP, Worsley KJ, Poline J-P, Frith CD, Frackowiak RSJ (1994) Statistical parametric maps in functional imaging: a general linear approach. Hum Brain Mapp 2:189–210. 10.1002/hbm.460020402

[B26] Glasser MF, et al. (2016) A multi-modal parcellation of human cerebral cortex. Nature 536:171–178. 10.1038/nature18933 27437579 PMC4990127

[B27] Gonzalez-Castillo J, Handwerker DA, Robinson ME, Hoy CW, Buchanan LC, Saad ZS, Bandettini PA (2014) The spatial structure of resting state connectivity stability on the scale of minutes. Front Neurosci 8:138. 10.3389/fnins.2014.00138 24999315 PMC4052097

[B28] Groen IIA, Dekker TM, Knapen T, Silson EH (2021) Visuospatial coding as ubiquitous scaffolding for human cognition. Trends Cogn Sci:S1364661321002813. 10.1016/j.tics.2021.10.011 34799253

[B29] Han LT, Epstein RA (2026) Distinct mechanisms for panoramic and landmark-based view integration in human place-selective cortex. J Neurosci 46:e0187252025. 10.1523/JNEUROSCI.0187-25.2025 41475765 PMC12873635

[B30] Hasson U, Levy I, Behrmann M, Hendler T, Malach R (2002) Eccentricity bias as an organizing principle for human high-order object areas. Neuron 34:479–490. 10.1016/S0896-6273(02)00662-1 11988177

[B31] Huffman DJ, Ekstrom AD (2019) A modality-independent network underlies the retrieval of large-scale spatial environments in the human brain. Neuron 104:611–622. 10.1016/j.neuron.2019.08.012 31540825 PMC6842116

[B32] Jo HJ, Gotts SJ, Reynolds RC, Bandettini PA, Martin A, Cox RW, Saad ZS (2013) Effective preprocessing procedures virtually eliminate distance-dependent motion artifacts in resting state FMRI. J Appl Math 2013:935154. 10.1155/2013/935154 PMC3886863 24415902

[B33] Julian JB, Ryan J, Hamilton RH, Epstein RA (2016) The occipital place area is causally involved in representing environmental boundaries during navigation. Curr Biol 26:1104–1109. 10.1016/j.cub.2016.02.066 27020742 PMC5565511

[B34] Kamps FS, Julian JB, Kubilius J, Kanwisher N, Dilks DD (2016) The occipital place area represents the local elements of scenes. Neuroimage 132:417–424. 10.1016/j.neuroimage.2016.02.062 26931815 PMC4872505

[B35] Kennedy B, Malladi SN, Tootell RB, Nasr S (2024) A previously undescribed scene-selective site is the key to encoding ego-motion in naturalistic environments. eLife 13:RP91601. 10.7554/eLife.91601 38506719 PMC10954307

[B36] Kundu P, Inati SJ, Evans JW, Luh WM, Bandettini PA (2012) Differentiating BOLD and non-BOLD signals in fMRI time series using multi-echo EPI. Neuroimage 60:1759–1770. 10.1016/j.neuroimage.2011.12.028 22209809 PMC3350785

[B37] Kundu P, Brenowitz ND, Voon V, Worbe Y, Vértes PE, Inati SJ, Saad ZS, Bandettini PA, Bullmore ET (2013) Integrated strategy for improving functional connectivity mapping using multiecho fMRI. Proc Natl Acad Sci U S A 110:16187–16192. 10.1073/pnas.1301725110 24038744 PMC3791700

[B38] Lescroart MD, Gallant JL (2019) Human scene-selective areas represent 3D configurations of surfaces. Neuron 101:178–192.e7. 10.1016/j.neuron.2018.11.004 30497771

[B39] Lu Z, Julian JB, Aguirre GK, Epstein RA (2025) A neural compass in the human brain during naturalistic virtual navigation. J Neurosci 45:e1765242025. 10.1523/JNEUROSCI.1765-24.2025 40825653 PMC12369924

[B40] Marchette SA, Vass LK, Ryan J, Epstein RA (2014) Anchoring the neural compass: coding of local spatial reference frames in human medial parietal lobe. Nat Neurosci 17:1598–1606. 10.1038/nn.3834 25282616 PMC4309016

[B41] Marchette SA, Vass LK, Ryan J, Epstein RA (2015) Outside looking in: landmark generalization in the human navigational system. J Neurosci 35:14896–14908. 10.1523/JNEUROSCI.2270-15.2015 26538658 PMC4635136

[B42] Mynick A, Steel A, Jayaraman A, Botch TL, Burrows A, Robertson CE (2025) Memory-based predictions prime perceptual judgments across head turns in immersive, real-world scenes. Curr Biol 35:121–130.e6. 10.1016/j.cub.2024.11.024 39694030 PMC11831858

[B43] Naveilhan C, Zory R, Gramann K, Ramanoël S (2025) Theta activity supports landmark-based correction of naturalistic human path integration. J Neurosci 45:e1005252025. 10.1523/JNEUROSCI.1005-25.2025 41006061 PMC12590116

[B44] Olszowy W, Aston J, Rua C, Williams GB (2019) Accurate autocorrelation modeling substantially improves fMRI reliability. Nat Commun 10:1220. 10.1038/s41467-019-09230-w 30899012 PMC6428826

[B45] Park S, Chun MM (2009) Different roles of the parahippocampal place area (PPA) and retrosplenial cortex (RSC) in panoramic scene perception. Neuroimage 47:1747–1756. 10.1016/j.neuroimage.2009.04.058 19398014 PMC2753672

[B46] Persichetti AS, Dilks DD (2016) Perceived egocentric distance sensitivity and invariance across scene-selective cortex. Cortex 77:155–163. 10.1016/j.cortex.2016.02.006 26963085 PMC4808380

[B47] Persichetti AS, Dilks DD (2018) Dissociable neural systems for recognizing places and navigating through them. J Neurosci 38:10295–10304. 10.1523/JNEUROSCI.1200-18.2018 30348675 PMC6596208

[B48] Persichetti AS, Dilks DD (2019) Distinct representations of spatial and categorical relationships across human scene-selective cortex. Proc Natl Acad Sci U S A 116:21312–21317. 10.1073/pnas.1903057116 31570605 PMC6800382

[B49] Petersen SE, Seitzman BA, Nelson SM, Wig GS, Gordon EM (2024) Principles of cortical areas and their implications for neuroimaging. Neuron 112:2837–2853. 10.1016/j.neuron.2024.05.008 38834069 PMC13339775

[B50] Pitcher D, Dilks DD, Saxe RR, Triantafyllou C, Kanwisher N (2011) Differential selectivity for dynamic versus static information in face-selective cortical regions. Neuroimage 56:2356–2363. 10.1016/j.neuroimage.2011.03.067 21473921

[B51] Poldrack RA, et al. (2015) Long-term neural and physiological phenotyping of a single human. Nat Commun 6:8885. 10.1038/ncomms9885 26648521 PMC4682164

[B52] Power JD, Barnes KA, Snyder AZ, Schlaggar BL, Petersen SE (2012) Spurious but systematic correlations in functional connectivity MRI networks arise from subject motion. Neuroimage 59:2142–2154. 10.1016/j.neuroimage.2011.10.018 22019881 PMC3254728

[B53] Power JD, Plitt M, Laumann TO, Martin A (2017) Sources and implications of whole-brain fMRI signals in humans. Neuroimage 146:609–625. 10.1016/j.neuroimage.2016.09.038 27751941 PMC5321814

[B54] Power JD, Plitt M, Gotts SJ, Kundu P, Voon V, Bandettini PA, Martin A (2018) Ridding fMRI data of motion-related influences: removal of signals with distinct spatial and physical bases in multiecho data. Proc Natl Acad Sci U S A 115:E2105–E2114. 10.1073/pnas.1720985115 29440410 PMC5834724

[B55] Rademaker RL, Chunharas C, Serences JT (2019) Coexisting representations of sensory and mnemonic information in human visual cortex. Nat Neurosci 22:1336–1344. 10.1038/s41593-019-0428-x 31263205 PMC6857532

[B56] Reynolds RC, Glen DR, Chen G, Saad ZS, Cox RW, Taylor PA (2024) Processing, evaluating, and understanding FMRI data with afni_proc.py. Imaging Neurosci 2:1–52. 10.1162/imag_a_00347 PMC11576932 39575179

[B57] Robertson CE, Hermann KL, Mynick A, Kravitz DJ, Kanwisher N (2016) Neural representations integrate the current field of view with the remembered 360° panorama in scene-selective cortex. Curr Biol 26:2463–2468. 10.1016/j.cub.2016.07.002 27618266

[B58] Rust NC, Palmer SE (2021) Remembering the past to see the future. Annu Rev Vis Sci 7:349–365. 10.1146/annurev-vision-093019-112249 34270350 PMC9751846

[B59] Saad ZS, Gotts SJ, Murphy K, Chen G, Jo HJ, Martin A, Cox RW (2012) Trouble at rest: how correlation patterns and group differences become distorted after global signal regression. Brain Connect 2:25–32. 10.1089/brain.2012.0080 22432927 PMC3484684

[B60] Saad ZS, Reynolds RC (2012) SUMA. Neuroimage 62:768–773. 10.1016/j.neuroimage.2011.09.016 21945692 PMC3260385

[B63] Scrivener CL, Silson EH (2025) Opponent visuospatial coding structures responses during memory recall and visual perception in medial parietal cortex. Imaging Neurosci 3:imag_a_00507. 10.1162/imag_a_00507 PMC12319830 40800799

[B61] Scrivener CL, Zamboni E, Morland AB, Silson EH (2024) Retinotopy drives the variation in scene responses across visual field map divisions of the occipital place area. J Vis 24:10. 10.1167/jov.24.8.10 PMC11343012 39167394

[B62] Scrivener CL, Teed JA, Silson EH (2025) Visual imagery of familiar people and places in category selective cortex. Neurosci Conscious 2025:niaf006. 10.1093/nc/niaf006 40241880 PMC12003044

[B64] Silson EH, Chan AWY, Reynolds RC, Kravitz DJ, Baker CI (2015) A retinotopic basis for the division of high-level scene processing between lateral and ventral human occipitotemporal cortex. J Neurosci 35:11921–11935. 10.1523/JNEUROSCI.0137-15.2015 26311774 PMC4549403

[B65] Silson EH, Groen IIA, Kravitz DJ, Baker CI (2016a) Evaluating the correspondence between face-, scene-, and object-selectivity and retinotopic organization within lateral occipitotemporal cortex. J Vis 16:14. 10.1167/16.6.14 PMC4898275 27105060

[B66] Silson EH, Steel AD, Baker CI (2016b) Scene-selectivity and retinotopy in medial parietal cortex. Front Hum Neurosci 10:412. 10.3389/fnhum.2016.00412 27588001 PMC4988988

[B67] Silson EH, Gilmore AW, Kalinowski SE, Steel A, Kidder A, Martin A, Baker CI (2019a) A posterior–anterior distinction between scene perception and scene construction in human medial parietal cortex. J Neurosci 39:705–717. 10.1523/JNEUROSCI.1219-18.2018 30504281 PMC6343650

[B68] Silson EH, Steel A, Kidder A, Gilmore AW, Baker CI (2019b) Distinct subdivisions of human medial parietal cortex support recollection of people and places. eLife 8:e47391. 10.7554/eLife.47391 31305238 PMC6667275

[B69] Smith SM, et al. (2013) Functional connectomics from resting-state fMRI. Trends Cogn Sci 17:666–682. 10.1016/j.tics.2013.09.016 24238796 PMC4004765

[B70] Srokova S, Hill PF, Rugg MD (2022) The retrieval-related anterior shift is moderated by age and correlates with memory performance. J Neurosci 42:1765–1776. 10.1523/JNEUROSCI.1763-21.2021 35017225 PMC8896550

[B71] Steel A, Thomas C, Trefler A, Chen G, Baker CI (2019) Finding the baby in the bath water – evidence for task-specific changes in resting state functional connectivity evoked by training. Neuroimage 188:524–538. 10.1016/j.neuroimage.2018.12.038 30578926

[B72] Steel A, Billings MM, Silson EH, Robertson CE (2021) A network linking scene perception and spatial memory systems in posterior cerebral cortex. Nat Commun 12:1–13. 10.1038/s41467-021-22848-z 33976141 PMC8113503

[B73] Steel A, Garcia BD, Silson EH, Robertson CE (2022) Evaluating the efficacy of multi-echo ICA denoising on model-based fMRI. Neuroimage 264:119723. 10.1016/j.neuroimage.2022.119723 36328274

[B74] Steel A, Garcia BD, Goyal K, Mynick A, Robertson CE (2023) Scene perception and visuospatial memory converge at the anterior edge of visually responsive cortex. J Neurosci 43:5723. 10.1523/JNEUROSCI.2043-22.2023 37474310 PMC10401646

[B75] Steel A, Angeli PA, Silson EH, Robertson CE (2024a) Retinotopic coding organizes the interaction between internally and externally oriented brain networks. bioRxiv:2024.09.25.615084.

[B76] Steel A, Silson EH, Garcia BD, Robertson CE (2024b) A retinotopic code structures the interaction between perception and memory systems. Nat Neurosci 27:339–347. 10.1038/s41593-023-01512-3 38168931 PMC10923171

[B77] Steel A, Prasad D, Garcia BD, Robertson CE (2025) Relating scene memory and perception activity to functional properties, networks, and landmarks of posterior cerebral cortex—a probabilistic atlas. J Neurosci 45:e0028252025. 10.1523/JNEUROSCI.0028-25.2025 40300833 PMC12139592

[B78] Sugiura M, Shah NJ, Zilles K, Fink GR (2005) Cortical representations of personally familiar objects and places: functional organization of the human posterior cingulate cortex. J Cogn Neurosci 17:183–198. 10.1162/0898929053124956 15811232

[B79] Sulpizio V, Fattori P, Pitzalis S, Galletti C (2023) Functional organization of the caudal part of the human superior parietal lobule. Neurosci Biobehav Rev 153:105357. 10.1016/j.neubiorev.2023.105357 37572972

[B80] Sun L, Frank SM, Epstein RA, Tse PU (2021) The parahippocampal place area and hippocampus encode the spatial significance of landmark objects. Neuroimage 236:118081. 10.1016/j.neuroimage.2021.118081 33882351

[B81] Suzuki S, Kamps FS, Dilks DD, Treadway MT (2021) Two scene navigation systems dissociated by deliberate versus automatic processing. Cortex 140:199–209. 10.1016/j.cortex.2021.03.027 33992908 PMC8608425

[B82] Van Assche M, Kebets V, Vuilleumier P, Assal F (2014) Functional dissociations within posterior parietal cortex during scene integration and viewpoint changes. Cereb Cortex:bhu215. 10.1093/cercor/bhu215 25246508

[B83] Wang L, Mruczek REB, Arcaro MJ, Kastner S (2015) Probabilistic maps of visual topography in human cortex. Cereb Cortex 25:3911–3931. 10.1093/cercor/bhu277 25452571 PMC4585523

[B84] Wolbers T, Büchel C (2005) Dissociable retrosplenial and hippocampal contributions to successful formation of survey representations. J Neurosci 22:2711–2717. 10.1523/JNEUROSCI.4705-04.2005 PMC6724902 15800188

[B85] Yoon HK, Jung Y, Persichetti AS, Dilks DD (2025) A scene-selective region in the superior parietal lobule for visually guided navigation. Cereb Cortex 35:bhaf082. 10.1093/cercor/bhaf082 40264261 PMC12014905

[B86] Yoon HK, Jung Y, Dilks DD (2026) Dissociable systems for visually guided navigation versus reaching and grasping in human parietal cortex. Cereb Cortex 36:bhag050. 10.1093/cercor/bhag050 42063373 PMC13133554

[B87] Zamboni E, Lowndes R, Aveyard R, Scrivener CL, Teed JA, Ma Y, Morland AB, Silson EH (2026) Early spatiotemporal dynamics of navigational affordance coding in the dorsal visual cortex. Nat Commun 17:1361. 10.1038/s41467-025-68111-7 41507190 PMC12876848

[B88] Zhang T, Meschke EX, Gallant J (2025) A map of the cortical functional network mediating naturalistic navigation. Available at: http://biorxiv.org/lookup/doi/10.64898/2025.12.16.694742 [Accessed April 26, 2026].

